# Multiple Reaction Monitoring (MRM)-Based Targeted Kidney Metabolite Profiling of a Mouse Model of Hyperuricemia

**DOI:** 10.3390/metabo16060362

**Published:** 2026-05-27

**Authors:** Hailong Li, Tingting Tang, Qingli Zhang, Tingting Song, Zichu Zhao, Lei Zhu, Qu Chen, Haili Zhang, Yan Zhang, Jingjing Kong

**Affiliations:** Department of Medicine, Qingdao Binhai University, 425 West Jialing River Road, Qingdao 266555, China; tangtingting@qdbhu.edu.cn (T.T.); zhangqingli@qdbhu.edu.cn (Q.Z.); songtingting@qdbhu.edu.cn (T.S.); zhaozichu@qdbhu.edu.cn (Z.Z.); zhulei@qdbhu.edu.cn (L.Z.); chenqu@qdbhu.edu.cn (Q.C.); zhanghaili@qdbhu.edu.cn (H.Z.); zhangyan@qdbhu.edu.cn (Y.Z.)

**Keywords:** hyperuricemia, multiple reaction monitoring, targeted metabolomics, animal model, chronic urate nephropathy

## Abstract

**Background/Objectives:** Chronic urate nephropathy (CUN), also referred to as gouty nephropathy, represents a severe renal disease primarily precipitated by long-term hyperuricemia (HUA) and gout. However, the precise molecular mechanisms underlying its pathogenesis remain poorly understood. The present study was designed to explore these mechanisms from the perspective of targeted metabolomics. **Methods:** The HUA mice constructed by urate oxidase (Uox) gene knockout (KO) and their corresponding wild-type controls were employed for the present study. Serum clinical biochemical parameters were determined, and renal histopathological changes were evaluated using hematoxylin-eosin (HE) staining and Masson’s trichrome staining. A targeted metabolomic strategy based on multiple reaction monitoring (MRM) was utilized to profile the renal metabolic landscape of Uox-KO mice, and potential metabolic biomarkers for CUN were identified via multivariate data analysis. **Results:** Clinical biochemical analysis revealed a significant elevation in serum uric acid, creatinine, and urea nitrogen levels in Uox-KO mice compared with control mice. Histopathological observations confirmed a typical CUN phenotype in Uox-KO mice, characterized by renal tubular vacuolar degeneration and dilatation, desquamation of tubular epithelial cells into the lumen, neutrophil infiltration, glomerular crowding, and renal interstitial fibrosis. Metabolomic analysis identified a total of 291 differentially regulated metabolites in Uox-KO mice relative to control animals. These perturbed metabolites were involved in multiple key biochemical pathways, including amino acid biosynthesis, ABC transporter signaling pathway, purine metabolism, aminoacyl-tRNA biosynthesis, protein digestion and absorption, glycerophospholipid metabolism, and serotonergic synaptic transmission. Notably, pathological parameters, including biochemical measurements and histological observations, were significantly correlated with key differential metabolites associated with CUN progression. Furthermore, eleven differential metabolites (pyroglutamic acid, fructose, riboflavin, dimethyl-L-arginine, glucaric acid, indoxyl sulfate, palmitoylethanolamide, trimethylamine N-oxide, 3-hydroxyanthranilic acid, spermidine, and hippuric acid) were identified as potential metabolic biomarkers for the diagnosis and prognosis of CUN. **Conclusions:** These findings illustrate that targeted tissue metabolomic analysis constitutes a powerful tool for deciphering the molecular mechanisms of diseases, thus offering novel insights into the pathogenesis of CUN.

## 1. Introduction

Uric acid (UA) is a weak organic acid primarily generated via hepatic purine metabolism. Due to multiple evolutionary mutations, higher primates, including humans, have lost uricase activity. As a result, UA—a relatively poorly water-soluble compound—serves as the terminal metabolite of both dietary and endogenous purine catabolism. In contrast, most other mammalian species further convert UA to allantoin, a more soluble derivative [1,2]. Elevated levels of serum UA (hyperuricemia, HUA), defined as levels exceeding 420 μmol/L, have become increasingly prevalent in recent years, representing a growing threat to global public health [3,4]. HUA is the primary pathogenic factor of gout, a common form of inflammatory arthritis. Gout affects 3–6% of males and 1–2% of females in Western industrialized countries [5]. In mainland China, its prevalence reaches 1.1% [3]. Clinically, gout manifests as acute attacks of severe joint pain, swelling, and functional impairment. Without timely intervention, it may progress to persistent joint lesions and irreversible structural damage [6]. Accumulating evidence also links HUA and gout to increased risks of hypertension, type 2 diabetes, obesity, metabolic syndrome, and cardiovascular diseases [6,7].

A growing body of research has confirmed a close association between HUA and kidney injury. HUA is now recognized as an independent risk factor for the onset and progression of chronic kidney disease (CKD) [8]. Clinical studies show that lowering serum UA levels can slow the progression of CKD. It also reduces cardiovascular complications in affected patients [9,10]. Moreover, HUA exacerbates the incidence and progression of diabetic kidney disease (DKD) [11] and acute kidney injury (AKI) [12]. To date, three major types of nephropathy have been directly linked to HUA: acute urate nephropathy [13], chronic UA nephropathy (CUN) [14], and UA nephrolithiasis [15]. Acute UA nephropathy is mainly characterized by anuric or oliguric AKI. It results from massive UA deposition and accumulation in renal tubules [16]. This condition frequently occurs in patients with acute leukemia or non-Hodgkin lymphoma. Such patients develop tumor lysis syndrome after cytotoxic chemotherapy [16]. CUN, also called gouty nephropathy, usually arises in individuals with long-term persistent HUA [14]. Pathologically, CUN lesions are driven by monosodium urate crystal deposition. These crystals accumulate in the acidic microenvironment of distal tubules, collecting ducts, and the renal interstitium [14]. UA nephrolithiasis involves the precipitation of anhydrous UA, UA dihydrate, sodium hydrogen urate monohydrate, or ammonium hydrogen urate in the kidney [15]. HUA, reduced urinary output, and acidic urine are well-established predisposing factors for UA nephrolithiasis [15,17]. HUA-induced nephropathy involves both crystal-dependent and crystal-independent mechanisms. The crystal-independent mechanisms include hemodynamic alterations and nonhemodynamic pathways. Hemodynamic alterations include elevated glomerular hydrostatic pressure and reduced renal perfusion [8]. Non-hemodynamic mechanisms encompass several pathological processes. These include the activation of vasoconstrictive signaling, the induction of vascular proliferation and inflammation, endothelial dysfunction, and the inhibition of endogenous vasodilatory pathways [8]. Despite these advances, the precise mechanisms remain incompletely understood.

The aim of metabolomics is to comprehensively profile low-molecular-weight metabolites through analyses of biological fluids, cells, tissues, or whole organisms under conditions of genetic modification, drug intervention, pathogen infection, or environmental stimulation [18,19,20,21]. Metabolomic approaches are generally categorized into untargeted and targeted strategies. Untargeted metabolomics enables the unbiased profiling of both known and unknown metabolites and is suitable for exploratory research without prior metabolic hypotheses [22,23]. Nevertheless, its major limitation is the low confidence of metabolite annotations, which stems from the technical bottlenecks intrinsic to compound identification [22]. Targeted metabolomics was developed to overcome this drawback. It focuses on the accurate identification and absolute quantification of a predefined panel of known metabolites. Multiple reaction monitoring (MRM) based on triple quadrupole mass spectrometry is a gold-standard technique for targeted metabolomics. MRM adopts a two-stage ion selection process: the precursor ion is screened in Q1, and the corresponding product ion is captured in Q3. Specific Q1–Q3 transitions allow for the structure-specific and high-accuracy quantification of individual metabolites [24,25]. Benefiting from high selectivity, superior sensitivity, and excellent reproducibility, MRM-based targeted metabolomics has been widely applied in biomarker and mechanistic explorations of various renal diseases. These include CKD, DKD, AKI, and IgA nephropathy (IgAN) [26,27,28,29,30,31]. For example, Xia et al. quantified 21 plasma metabolites in patients with DKD and identified adenosine, inosine, UA, xanthine, and creatinine (CRE) as potential diagnostic biomarkers [26]. Another MRM-based study quantified 545 lipid species in patients with IgAN and healthy controls, revealing eight upregulated triacylglycerols as independent risk factors for IgAN progression [30]. In addition, a combined MRM metabolomics and proteomics analysis uncovered a novel mechanism of wasp sting-induced AKI. This mechanism is mediated by rhabdomyolysis and intravascular hemolysis [31]. However, MRM-based targeted metabolomics has rarely been applied to investigate metabolic alterations in urate nephropathy (UN).

In our previous work, we established a mouse model of CUN by knocking out (KO) the uricase oxidase (Uox) gene. The plasma and kidney metabolic profiles were preliminarily characterized using untargeted metabolomics analysis [20]. In the present study, we performed, for the first time, an MRM-based targeted metabolomics analysis to identify metabolic signatures in the kidneys of Uox-KO mice and further explored candidate biomarkers for CUN. These findings may help elucidate the molecular mechanisms underlying CUN. With further investigation, this work may also facilitate the development of metabolite-based strategies for the prevention and treatment of CUN.

## 2. Materials and Methods

### 2.1. Bioethics, Experimental Design, and Sample Collection

This study was approved by the Animal Research Ethics Committee of Qingdao Binhai University (Approval No.: 2024-41; Approval date: 15 October 2024). The research was conducted in compliance with the ARRIVE guidelines, the UK Guidance on the Operation of the Animals (Scientific Procedures) Act 1986, and associated regulations. All animal procedures were performed by certified personnel in the Experimental Animal Facility of Qingdao Binhai University.

Heterozygous HUA mice (Uox^+^/^−^) were generously provided by Professor Changgui Li (The Affiliated Hospital of Qingdao University). These mice were generated using a method described previously [32]. Homozygous Uox-KO mice were obtained from heterozygous crosses. The present study used eight-week-old male whole-body Uox-KO mice (*n* = 6) and age- and sex-matched wild-type (WT) mice (serving as controls) were used in the present study. Genotyping of Uox-KO mice was confirmed via polymerase chain reaction (PCR) using genomic DNA extracted from tail snips. Animals were housed in a specific pathogen-free (SPF) animal facility under controlled environmental conditions. These conditions included a 12 h light/dark cycle, a temperature of 22–24 °C, and a relative humidity of 40–60%. Standard laboratory chow and sterile water were available ad libitum.

Mice were euthanized via CO_2_ inhalation at a displacement rate of 25% volume per minute. Blood samples were collected immediately post-euthanasia via retro-orbital plexus bleeding. Serum was isolated by centrifugation at 2000× *g* for 10 min at 4 °C. The serum samples were then stored at −20 °C until biochemical analysis. Kidney tissues were harvested promptly and divided into two aliquots. One aliquot was snap-frozen in liquid nitrogen within 10 min post-mortem. This aliquot was stored at −80 °C for metabolomic analysis. The other aliquot was fixed in 10% neutral buffered formalin (Solarbio Co., Ltd., Beijing, China) at room temperature (20–25 °C) for 24 h. After fixation, the tissue was preserved in 70% ethanol until histopathological examination.

### 2.2. Biochemistry Analysis of the Serum

Serum biochemical parameters were measured using a Cobas c501 analyzer (Roche Diagnostics, Basel, Switzerland). The parameters included UA, CRE, urea nitrogen (BUN), glucose (GLU), triglycerides (TG), total cholesterol (TC), alanine transaminase (ALT), aspartate transaminase (AST), high-density lipoprotein (HDL), low-density lipoprotein (LDL), and albumin (ALB).

### 2.3. Histopathological Analysis of the Kidneys

Histological analysis, including hematoxylin-eosin (H & E) staining and Masson’s trichrome (MT) staining of kidney tissues, was performed in accordance with previously established protocols [33]. Briefly, fixed kidney tissue aliquots were embedded in paraffin to prepare paraffin blocks. Serial 5 µm-thick sections were cut using a rotary microtome (Leica Biosystems, Nussloch, Germany) and mounted on glass slides for histopathological evaluation. The sections were then subjected to H & E and MT staining following standard procedures. Pathological changes in the kidneys were observed and imaged using a Carl Zeiss Axio Imager M2 microscope (Carl Zeiss AG, Oberkochen, Germany). Renal tubular injury was semi-quantitatively assessed using a tubular injury score (TIS). The score ranged from 0 to 3, where 0 = normal tubular structure, 1 = mild tubular damage, 2 = moderate tubular damage, and 3 = severe tubular damage [34]. All histological evaluations were performed by two independent pathologists who were blinded to the experimental groups to ensure objectivity.

### 2.4. MRM Targeted Metabolomic Analysis

The MRM-based targeted metabolomic analysis used in this study was conducted by BioProfile biotechnology company (Shanghai, China). The analysis followed established methods with minor modifications [35,36].

#### 2.4.1. Chemicals and Reagents

All reagents used for metabolomic analysis were of liquid chromatography-mass spectrometry (LC-MS) grade. Methanol and acetonitrile were purchased from Merck KGaA (Darmstadt, Germany). Ammonium acetate was obtained from Sigma-Aldrich Co., LLC (St. Louis, MO, USA). L-glutamate-D5 was purchased from Toronto Research Chemicals Inc. (Toronto, ON, Canada). Formic acid was acquired from MilliporeSigma (Burlington, MA, USA). Ultrapure water was prepared using a Milli-Q Integral System (MilliporeSigma, Burlington, MA, USA).

#### 2.4.2. Sample Preparation for Metabolite Profiling

Metabolite extraction from kidney tissues was performed as follows. Briefly, 30 mg of snap-frozen kidney tissue was thawed on ice. The tissue was then mixed with 200 μL of precooled ddH_2_O and 800 μL of methanol/acetonitrile mixture (*v*/*v* = 1:1). Tissue homogenization was achieved using a TissueLyser bead-mill homogenizer (QIAGEN, Hilden, Germany). After homogenization, the mixture was sonicated in an ice bath for 1 h. It is then incubated at −20 °C for 2 h to facilitate protein precipitation. The samples were then centrifuged at 16,000× *g* for 30 min at 4 °C. The supernatants were carefully collected. An equal volume of L-glutamate-D5 internal standard (0.01 μg/μL) was added to the supernatants. The mixture was then concentrated to dryness under vacuum. The dried extracts were reconstituted in 100 μL of methanol/ddH_2_O (*v*/*v* = 1:1). The reconstituted samples were centrifuged at 16,000× *g* for 15 min at 4 °C to remove residual precipitates. The final supernatants were transferred to LC-MS sampling vials for subsequent analysis.

To ensure data quality for metabolic profiling, quality control (QC) samples were prepared by pooling aliquots of all experimental samples, thus ensuring they were representative of the entire sample set under analysis. QC samples were prepared and analyzed using the same procedures as the experimental samples in each batch, and were used for data normalization. Additionally, these QC samples were employed to monitor system stability, reproducibility, and analytical consistency throughout the experiment, further validating the reliability of the metabolomic data.

#### 2.4.3. LC-MS/MS Analysis

The LC/MS portion of the platform was based on a Shimadzu Nexera X2 LC-30AD system (Shimadzu Corporation, Kyoto, Japan) equipped with an Acquity ultraperformance liquid chromatography (UPLC) HSS T3 column (2.1 × 50 mm, particle size 1.8 μm, Waters Corporation, Milford, MA, USA) and a 5500 QTRAP triple quadrupole mass spectrometer (AB Sciex Instruments, Foster City, CA, USA). Metabolites were detected in electrospray ionization (ESI) positive (ESI+) and negative (ESI–) modes. A 5 μL aliquot of each sample was injected sequentially via the LC autosampler. The Acquity UPLC HSS T3 column was maintained at 40 °C with a constant flow rate of 200 μL/min. Gradient elution was employed for compound separation, using 0.1% formic acid aqueous solution (solvent A) and 100% acetonitrile (solvent B) as the mobile phases. The gradient elution for the positive-ionization mode was conducted as follows: 0% solvent B for 0–2.5 min; 0–30% solvent B for 2.5–9 min; 30–100% solvent B for 9–10 min; 100% solvent B for 10–15.4 min; 100–0% solvent B for 15.4–15.5 min; and 0% solvent B for 15.5–18 min (column re-equilibration). The gradient elution for the negative-ionization mode was conducted as follows: 0% solvent B for 0–2 min; 0–20% solvent B for 2–2.5 min; 20–40% solvent B for 2.5–3.5 min; 40–50% solvent B for 3.5–4.5 min; 50–100% solvent B for 4.5–10 min; 100% solvent B for 10–15.4 min; 100–0% solvent B for 15.4–15.5 min; and 0% solvent B for 15.5–18 min (column re-equilibration). The MS conditions were set as follows. For negative-ionization mode: source temperature (ST), 550 °C; ion source gas1 (GAS1): 40 psi; ion source gas2 (GAS2), 50 psi; curtain gas (CUR): 35 psi; ion spray voltage floating (ISVF), −4500 V. For positive-ionization mode: ST, 550 °C; GAS1, 40 psi; GAS2, 50 psi; CUR, 35 psi; ISVF, 5500 V. Transitions were detected in MRM mode.

To construct the in-house metabolite MRM library, each metabolite standard (50 mg/mL) was analyzed by LC-MS/MS to obtain the optimal MRM transition parameters. Subsequently, the retention time (RT) of 625 metabolites was determined by individually measuring their corresponding MRM (Q1/Q3) transitions.

#### 2.4.4. Data Processing for Metabolomic Analysis

Raw MRM data files were processed using MultiQuant 3.0.2 software (AB Sciex). The processing steps included peak finding, alignment, extraction, and filtering. Low-quality ions with a relative standard deviation >30% in QC samples were filtered out. Metabolites were identified by comparing their RT and MS features with the in-house MRM library. They were assigned confidence levels 1–3 according to the Metabolomics Standard Initiative. Metabolite features were analyzed based on the measured LC-MS peak areas, which are proportional to feature concentration. The peak areas were then normalized using the internal standard L-glutamate-D5.

Multivariate analyses, including principal component analysis (PCA), partial least squares discriminant analysis (PLS-DA), and orthogonal PLS-DA (OPLS-DA), were employed to identify outliers and maximize the separation between groups. The OPLS-DA models were validated using permutation tests with 200 permutations. Relevant R^2^ and Q^2^ values were used to evaluate model stability and explanatory ability. PLS-DA models were regarded as valid only if Q^2^ > 0.5. In this study, differential metabolites were screened using the variable importance in the projection (VIP) threshold of the first two principal components from the OPLS-DA model, combined with univariate analysis of *p*-values. Metabolites that met the VIP threshold (VIP > 1) and passed the two-tailed unpaired *t*-test (*p* < 0.05) were identified as differential metabolites between the two groups. To identify perturbed biological pathways, the differential metabolites between Uox-KO mice and WT mice were mapped to the Kyoto Encyclopedia of Genes and Genomes (KEGG) pathway database and subjected to enrichment analysis. Hierarchical cluster analysis was used to reveal distinct metabolomic signatures between Uox-KO and WT mice. This analysis was based on the differentially abundant metabolites. Potential metabolic biomarkers for UN were predicted using receiver operating characteristic (ROC) curve analysis by calculating the area under the curve (AUC) of all differential metabolites in the kidney.

### 2.5. Statistical Analysis

Blood biochemical data were assessed for normality and homogeneity of variance. After these assumptions were met, an unpaired two-tailed *t*-test was performed to compare the biochemical differences between Uox-KO mice and WT mice. A two-tailed unpaired *t*-test was also used to analyze differential metabolites between the two groups. The false discovery rate (FDR) of the *p*-values was corrected using the Benjamini–Hochberg FDR algorithm. KEGG enrichment analyses were performed using Fisher’s exact test, with FDR correction applied for multiple testing. Correlations between identified differential metabolites and kidney functional parameters were investigated. These parameters included UA, CRE, BUN, and TIS. Pearson correlation was used for normally distributed variables, whereas Spearman correlation was used for non-parametric variables. All statistical analyses were carried out using SPSS 25.0 software. A *p*-value < 0.05 was considered statistically significant.

## 3. Results

### 3.1. Biochemistry and Histopathological Analysis

**Figure 1:**
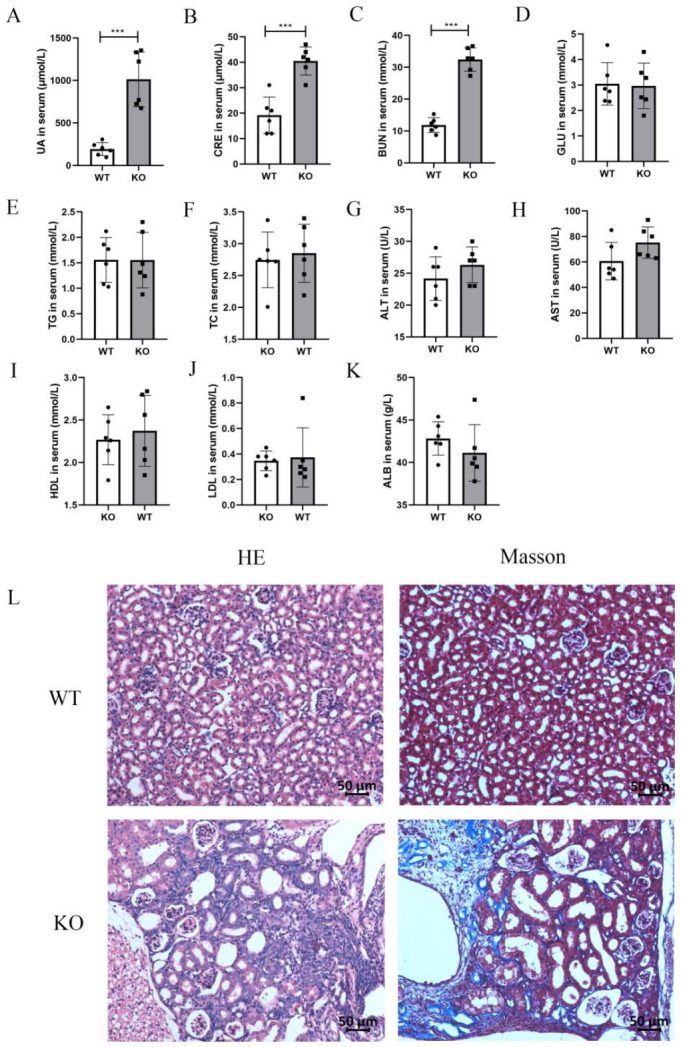
Biochemistry and histopathological analysis of the kidneys from the Uox-KO mice and WT mice. (**A**) The level of uric acid (UA) in Uox-KO mice and WT mice; (**B**) The level of creatinine (CRE) in Uox-KO mice and WT mice; (**C**) The level of urea nitrogen (BUN) in Uox-KO mice and WT mice; (**D**) The level of glucose (GLU) in Uox-KO mice and WT mice; (**E**) The level of triglycerides (TG) in Uox-KO mice and WT mice; (**F**) The level of total cholesterol (TC) in Uox-KO mice and WT mice; (**G**) The level of alanine aminotransferase (ALT) in Uox-KO mice and WT mice; (**H**) The level of aspartate aminotransferase (AST) in Uox-KO mice and WT mice; (**I**) The level of high-density lipoprotein (HDL) in Uox-KO mice and WT mice; (**J**) The level of low-density lipoprotein (LDL) in Uox-KO mice and WT mice; (**K**) The level of albumin (ALB) in Uox-KO mice and WT mice; (**L**) Hematoxylin and eosin (H & E) pathological staining and Masson’s trichrome staining of the kidneys from Uox-KO and WT mice. The data are presented as means ± SD, *n* = 6 in each group mice aged from 8 weeks. *** *p* < 0.001.

As shown in Figure 1A–C, Uox-KO mice exhibited significant elevations in serum UA, CRE, and BUN compared with WT controls. In contrast, no significant differences were observed in other biochemical parameters between the two groups. These parameters included GLU, TG, TC, ALT, AST, HDL, LDL, and ALB (Figure 1D–K).

H & E and MT staining revealed a distinct CUN phenotype in Uox-KO mice. This phenotype was characterized by vacuolar degeneration and enlargement of renal tubules, desquamation of tubular epithelial cells into the lumen, neutrophil infiltration, glomerular crowding, and renal interstitial fibrosis (Figure 1L).

### 3.2. Evaluation of the Reproducibility of the Metabolomic Analysis Method

**Figure 2:**
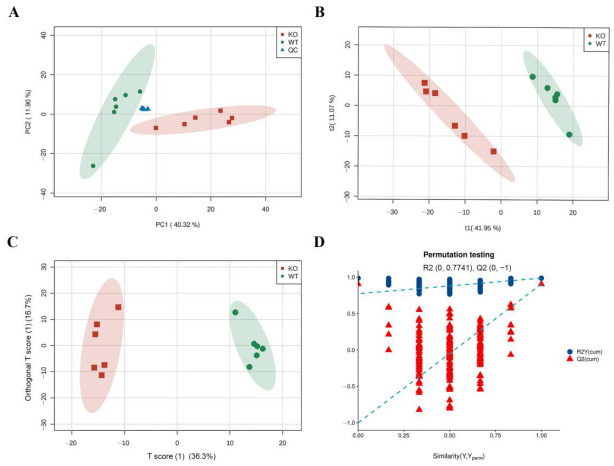
Multivariate statistical analysis of kidney metabolic profiling. (**A**) Principal component analysis (PCA) score plot from Uox-KO mice (Ko), WT mice (WT) and QCs. (**B**) Partial least squares discriminant analysis (PLS-DA) score plot from Uox-KO mice (Ko) and WT mice (WT). (**C**) OPLS-DA score plot from Uox-KO mice (KO) and WT mice (WT). (**D**) Cross-validation plot of the OPLS-DA model with a permutation test repeated 200 times. The blue dashed lines represent the corresponding fitted regression lines for observed and permuted R^2^ and Q^2^ values.

The stability of the metabolomic profiling system is essential for the relative quantification of spectra among different samples. Therefore, we used QC samples to evaluate the stability and reproducibility of the analytical method. Typical base peak ion chromatograms of QC samples in positive and negative ion modes showed efficient overlap across all sample types, with negligible fluctuations in RT and peak response intensity (Figure S1). Furthermore, the percentages of features with a coefficient of variation (CV) ≤ 0.5 and ≤0.3 in QC samples exceeded 90% (Figure S2). In addition, PCA score plots demonstrated that all kidney-derived QC samples were tightly clustered (Figure 2A). Collectively, these results indicate high stability and reproducibility of the metabolomic analysis method employed in the present study.

### 3.3. Multivariate Analysis

PCA was used to detect the overall metabolic differences between Uox-KO mice and controls. The PCA score plot of kidney tissues is shown in Figure 2A, with two principal components identified for the two groups. The PCA score plot clearly distinguished the two groups (Figure 2A). Furthermore, we used a PLS-DA classification model to distinguish the two groups, and the two groups could also be successfully discriminated by the PLS-DA model (Figure 2B). Three principal components were obtained from the PLS-DA model with the following parameters: R^2^X = 0.62, R^2^Y = 0.998, and Q^2^ = 0.97 (Figure 2B). These parameters adequately demonstrated the metabolic differences between the two groups. To obtain more accurate results, we performed OPLS-DA to filter out orthogonal information unrelated to classification. Based on OPLS-DA, Uox-KO mice and the control group were discriminated with an R^2^X of 0.53, an R^2^Y of 0.988, and a Q^2^ of 0.907 (Figure 2C). A random permutation test with 200 permutations was conducted for OPLS-DA to further evaluate the robustness of this method. As shown in Figure 2D, the validation plots strongly confirmed that the models were not overfitted, as the Q^2^ regression lines had negative intercepts (intercepts: Q^2^ 0.0, −1).

### 3.4. Metabolomic Profiling from Kidney Tissues

**Figure 3:**
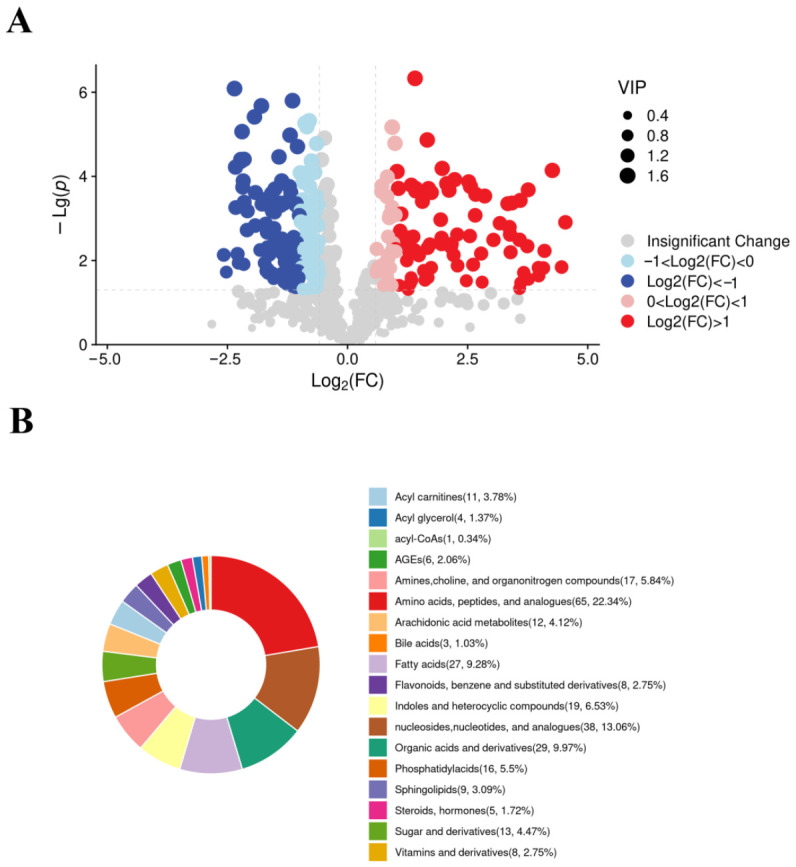
Volcano plots and classification of differential metabolites between Uox-Ko and WT mice. (**A**) Volcano plots of the differentially abundant metabolites from the kidneys of the Uox-KO mice. The horizontal axis denotes the log2-transformed fold change (log2FC) of metabolites between Uox-KO mice and WT mice, while the vertical axis represents the negative log10-transformed *p* value (-Lg(*p*)), reflecting the statistical significance of differences. Each dot in the plot corresponds to an individual metabolite. Dots of different colors represent different log2FC values, and the size of each dot is proportional to its VIP value. A gray dashed line parallel to the X-axis indicates the significance threshold of *p* = 0.05. Two additional gray dashed lines parallel to the Y-axis indicate fold change thresholds of FC = 0.5 (left) and FC = 2 (right) (**B**) Classification of differential metabolites between Uox-Ko and WT mice.

After MRM metabolomic analysis, a total of 291 metabolites were quantified to display differential abundances in the kidneys of Uox-KO mice (Figure 3A). In the ESI+ mode, 158 metabolites exhibited differential abundances; among these, 64 and 94 metabolites showed significantly increased and decreased levels, respectively, in Uox-KO mice (Table S1). Detailed information regarding these differential metabolites is provided in Table S1. Meanwhile, in the ESI–mode, 133 metabolites displayed differential abundances, of which 43 and 90 metabolites exhibited markedly increased and decreased levels, respectively, in Uox-KO mice (Table S2).

These differential metabolites could be classified into 18 categories, including acyl carnitines (3.78%), acyl glycerol (1.37%), acyl-CoAs (0.34%), advanced glycation end products (AGEs, 2.06%), amines, choline, and organonitrogen compounds (5.84%), amino acids, peptides, and analogs (22.34%), arachidonic acid metabolites (4.12%), bile acids (1.03%), fatty acids (FA, 9.28%), flavonoids, benzene and substituted derivatives (2.75%), indoles and heterocyclic compounds (6.53%), nucleosides, nucleotides, and analogues (13.06%), organic acids and derivatives (9.97%), phosphatidylacids (5.5%), sphingolipids (3.09%), steroids and hormones (1.72%), sugars and derivatives (4.47%), and vitamins and derivatives (2.75%) (Figure 3B).

**Figure 4:**
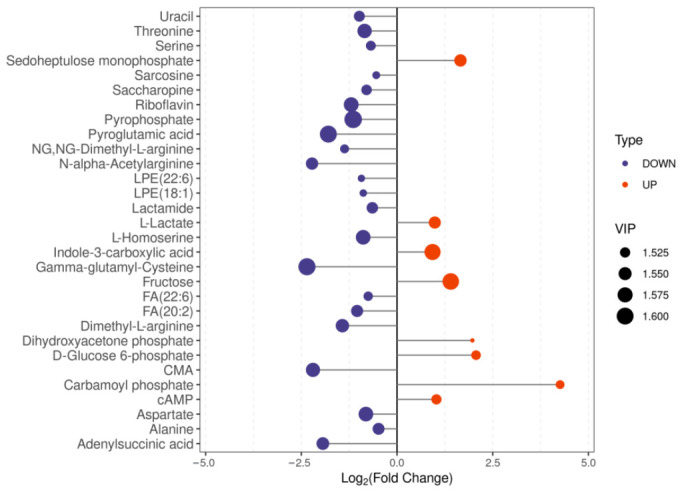
Bar chart of differential metabolites between Uox-KO mice and WT mice. Only the VIP top 30 differential metabolites are presented. The abscissa denotes the log-transformed fold change log2 (Fold Change), and the ordinate denotes metabolites. Blue and red dots on the left and right sides indicate downregulated and upregulated differential metabolites, respectively. The size of the dots represents the VIP value: the larger the dot, the higher the VIP value, indicating greater importance of the variable.

**Figure 5:**
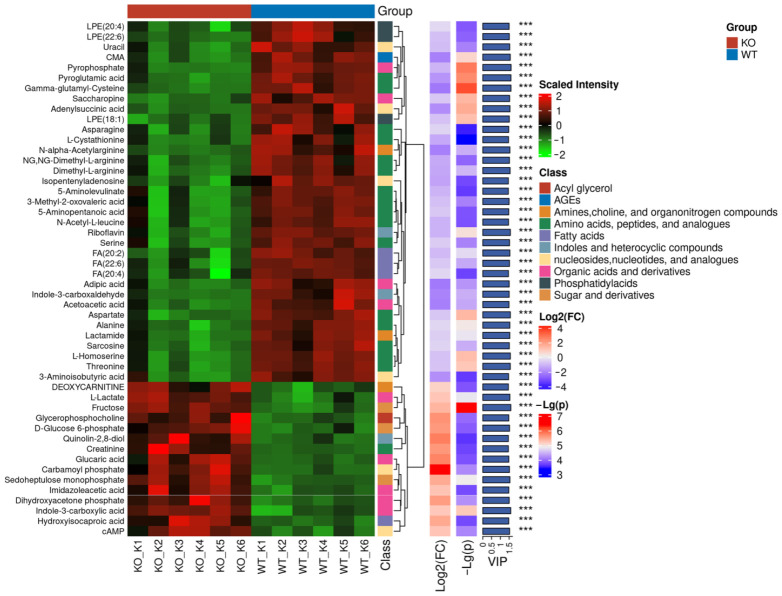
Z score heatmap of the significantly dysregulated metabolites from the kidneys of the Uox-KO mice. Only the VIP top 50 differential metabolites are presented. Each row represents an individual sample, and each column represents a metabolite. Scaled intensity indicates the expression level after standardization (the redder the color, the higher the expression level). Group refers to sample groups, and class refers to metabolite categories. *** *p* < 0.001.

The top 30 differential metabolites, ranked by VIP values, are listed below. These metabolites include: pyrophosphate, gamma-glutamyl-cysteine, pyroglutamic acid, fructose, indole-3-carboxylic acid, riboflavin, aspartate, L-homoserine, threonine, N7-carboxymethyl-arginine, dimethyl-L-arginine, adenyl-succinic acid, sedoheptulose monophosphate, N-alpha-acetyl-arginine, L-lactate, FA (20:2), alanine, lactamide, uracil, saccharopine, adenosine, 3′, 5′-cyclic AMP (cAMP), serine, D-glucose 6-phosphate, FA (22:6), NG, NG-dimethyl-L-arginine, carbamoyl phosphate, sarcosine, lysophosphatidylethanolamines (LPE) (18:1), LPE (22:6), and dihydroxyacetone phosphate (Figure 4). As shown in Figure 5, hierarchical clustering heatmap analysis of the top 50 VIP-ranked differential metabolites revealed distinct abundance patterns. Clear differences in relative abundances were observed between Uox-KO mice and WT mice.

### 3.5. Pathway Enrichment Analysis of the Differential Metabolites

**Figure 6:**
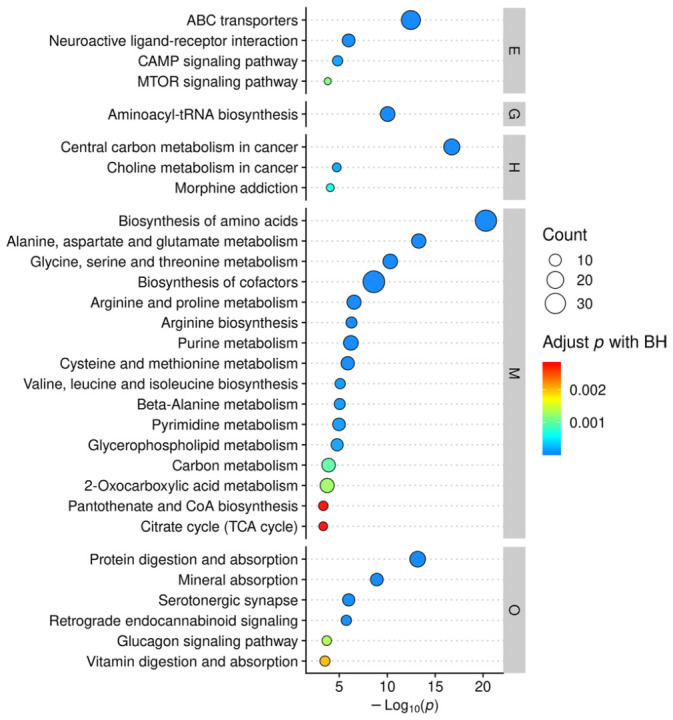
The main metabolic pathways changed in the Uox-KO mice. Only the top 30 differential metabolic pathways are presented. Pathway analysis was conducted based on the significantly altered metabolites between the Uox-KO and WT mice. The *X*-axis represents the negative logarithmic transformation of *p*-value, and the *Y*-axis represents the pathway name. Color gradient indicates the significance of the pathway ranked by *p*-value (blue: lower *p*-values and red: higher *p*-values). The size of the dot represents the number of different metabolites annotated to the pathway. The letters E, G, H, M, and O represent perturbed pathways in environmental information processing, genetic information processing, human diseases, metabolism, and organismal systems, respectively.

**Figure 7:**
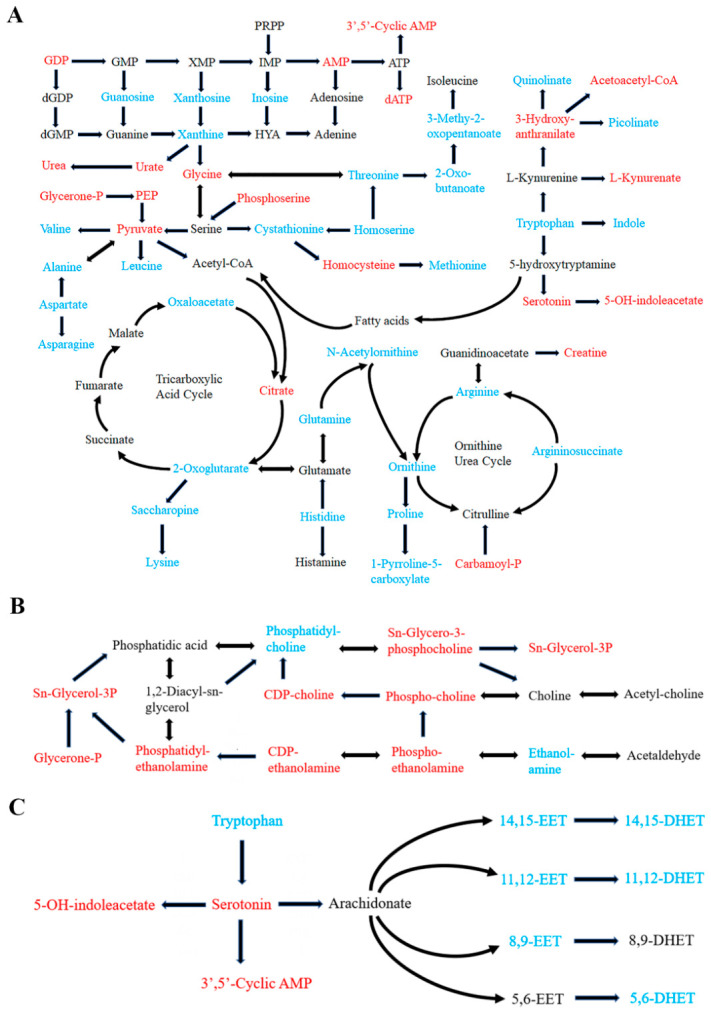
Map illustrating the predominantly disturbed pathways and the biochemical linkages among these metabolites in the Uox-KO mice. (**A**) The disturbed pathways involve purine metabolism, and amino acid biosynthesis and metabolism. AMP: adenosine 5′-monophosphate; ATP: adenosine 5′-triphosphate; dATP: deoxyadenosine 5′-triphosphate; dGDP: 2′-Deoxyguanosine 5′-diphosphate; dGMP: 2′-Deoxyguanosine 5′-monophosphate; GDP: guanosine 5′-diphosphate; GMP: guanosine 5′-phosphate; HYA: hypoxanthine; IMP: inosine monophosphate; PEP: phosphoenolpyruvate; PRPP: 5-Phosphoribosyl 1-pyrophosphate; XMP: xanthosine 5′-phosphate. (**B**) The disturbed pathways involving glycerophospholipid metabolism. (**C**) The disturbed pathways involving serotonergic synapse. DHET: dihydroxyeicosatrienoic acids; EET: epoxyeicosatrienoic acids. The metabolites marked in red font indicate increased levels in the Uox-KO mice compared with that of the controls, while the metabolites marked in blue font indicate decreased levels in the Uox-KO mice compared with that of the controls. Solid black arrows denote metabolic connections between metabolites.

Differential metabolites in the kidneys between Uox-KO mice and controls were mapped to the KEGG pathways and used for enrichment analysis. The main altered pathways included biosynthesis of amino acids, ABC transporters, purine metabolism, aminoacyl-tRNA biosynthesis, protein digestion and absorption, glycerophospholipid metabolism, and serotonergic synapse (Figure 6). Purine metabolism was found to be disturbed in Uox-KO mice, with significantly increased levels of AMP, dATP, cAMP, GDP, urate, and urea, and decreased levels of guanosine, inosine, xanthine, and xanthosine (Figure 6 and Figure 7A). Metabolic dysfunctions in amino acid biosynthesis were also observed in the kidneys of Uox-KO mice, in which levels of carbamoyl-P, citrate, glycerone-P, glycine, homocysteine, phosphoenolpyruvate, phosphoserine, and pyruvate were markedly increased, while levels of alanine, asparagine, aspartate, cystathionine, glutamine, histidine, homoserine, leucine, lysine, L-arginine, L-arginosuccinate, L-ornithine, L-proline, L-pyrroline-5-carboxylate, methionine, 3-methyl-2-oxopentanoate, N-acetylornithine, oxaloacetate, 2-oxo-butanoate, 2-oxoglutarate, saccharopine, threonine, and valine were markedly decreased (Figure 6 and Figure 7A). Tryptophan (TRP) metabolism was perturbed in Uox-KO mice, with dramatically increased levels of acetoacetyl-CoA, 3-hydroxy-anthranilate, L-kynurenate, serotonin, and 5-OH-indoleacetate (5-HIAA), and decreased levels of quinolinate, picolinate, TRP, and indole (Figure 7A). In addition, glycerophospholipid metabolism was also found to be disturbed in Uox-KO mice, with significantly increased levels of glycerone-P, sn-glycerol-3P, sn-glycero-3-phosphochine, CDP-choline, phospho-choline, phosphatidyl-ethanolamine, CDP-ethanolamine, and phospho-ethanolamine, and decreased levels of phosphatidyl-choline and ethanol-amine (Figure 7B). Moreover, metabolic pathways involving serotonergic synapse were dysregulated in Uox-KO mice, with obviously increased levels of cAMP, 5-HIAA, and serotonin, and decreased levels of TRP, 8,9-epoxyeicosatrienoic acids (EET), 11,12-EET, 14,15-EET, 5,6-dihydroxyeicosatrienoic acids (DHET), 11,12-DHET, and 14,15-DHET (Figure 7C).

### 3.6. Kidney Potential Metabolic Biomarkers for CUN

ROC curve analysis with AUC was performed for all differential metabolites in the kidneys to predict potential biomarkers for CUN. A total of 11 metabolites were identified by classical univariate ROC analyses with AUC = 1, including pyroglutamic acid, fructose, riboflavin, dimethyl-L-arginine, glucaric acid, indoxyl sulfate, palmitoylethanolamide, trimethylamine N-oxide, 3-hydroxyanthranilic acid, spermidine, and hippuric acid (Table 1). The correlations of these 11 metabolites with renal function parameters, including UA, CRE, BUN, and TIS, were further analyzed. All these metabolites (pyroglutamic acid, fructose, riboflavin, dimethyl-L-arginine, glucaric acid, indoxyl sulfate, palmitoylethanolamide, trimethylamine N-oxide, 3-hydroxyanthranilic acid, spermidine, and hippuric acid) were significantly associated with the renal functional parameters (*p* < 0.05) (Table 2).

## 4. Discussion

In this study, we used MRM-based targeted metabolomics to characterize the renal metabolic profile of Uox-KO mice. The Uox-KO mice displayed dramatically elevated serum UA, CRE, and BUN levels, as well as typical pathological characteristics of CUN. Multivariate analyses, including PCA, PLS-DA, and OPLS-DA, clearly distinguished Uox-KO mice from controls. These observations demonstrated the profound and, in some instances, tissue-specific metabolic impacts of CUN.

The metabolomic analysis of Uox-KO mouse kidneys revealed disturbances in multiple pathways, including the biosynthesis of amino acids, ABC transporters, purine metabolism, aminoacyl-tRNA biosynthesis, protein digestion and absorption, glycerophospholipid metabolism, and serotonergic synapse. These findings suggest that metabolic dysfunctions in these pathways may contribute to the CUN pathogenesis.

Purine metabolism generally consists of de novo synthesis, catabolism, and salvage pathways. The deregulation and abnormalities of purine metabolism are closely linked with neurodegenerative diseases, such as autism, Alzheimer’s disease, and Parkinson’s disease [37,38,39], as well as chronic inflammatory airway disease [40]. In this study, we detected disturbed purine metabolism in the kidneys of 8-week-old Uox-KO mice. Specifically, levels of AMP, dATP, cAMP, GDP, urate, and urea were significantly increased. In contrast, levels of guanosine, inosine, xanthine, and xanthosine were decreased (Figure 7A). These renal metabolic abnormalities are consistent with the results of a previous untargeted metabolomics analysis. That study reported elevated UA and 3′-AMP levels, and decreased inosine levels in 11- and 12-week-old mice [20]. Uox is a key enzyme in purine metabolism, which catalyzes the transformation of UA to allantoin. Evidently, the accumulation of UA and its downstream metabolite urea in the kidneys is a consequence of the loss of Uox activity. Purine bases can be recycled through salvage pathways to form their mononucleotides. This process involves several crucial enzymes, such as hypoxanthine-guanine phosphoribosyltransferase, adenine phosphoribosyltransferase, and adenosine kinase [41]. We hypothesize that UA accumulation in Uox-KO mice may inhibit feedback or activate these salvage pathway enzymes. This could explain the increased levels of AMP, dATP, cAMP, and GDP as well as the decreased levels of guanosine, inosine, xanthine, and xanthosine.

In this study, we identified disrupted amino acid biosynthesis in the kidneys of Uox-KO mice. Within the tricarboxylic acid (TCA) cycle, citrate levels were markedly elevated. Conversely, oxaloacetate and 2-oxoglutarate abundances were significantly reduced. The ornithine urea cycle also exhibited dysregulation: N-acetylornithine, L-ornithine, L-arginosuccinate, and L-arginine levels were all decreased (Figure 7A). The dramatically increased citrate levels in the TCA cycle of Uox-KO mice are consistent with previous studies [20]. In contrast, the decreased N-acetylornithine, L-ornithine, L-arginosuccinate, and L-arginine levels in the ornithine urea cycle of Uox-KO mice have not been reported previously. L-ornithine is produced by arginase II, reabsorbed along the proximal convoluted tubule, and transported by basolateral carriers [42]. Furthermore, L-ornithine can activate Ca^2+^ signaling to exert its protective function on human proximal tubular cells [43]. L-arginine is a semi-essential amino acid synthesized via the intestinal–renal axis in humans and most mammals [44]. L-arginine supplementation has beneficial effects in several models of CKDs, including renal ablation, ureteral obstruction, nephropathy secondary to diabetes, and salt-sensitive hypertension [45]. In addition, nephroprotective roles of L-arginine in AKI have been identified in rats and kidney transplantation patients [46,47]. Taken together, the decreased concentrations of L-ornithine and L-arginine in the kidneys of Uox-KO mice might indicate a reduction in their renal protective capacity.

Beyond the amino acids involved in the TCA and ornithine urea cycles, additional amino acids were dysregulated in Uox-KO mouse kidneys. Glycine, homocysteine, and phosphoserine levels were dramatically elevated. Conversely, alanine, asparagine, aspartate, cystathionine, glutamine, histidine, homoserine, leucine, lysine, L-proline, threonine, and valine levels were significantly reduced (Figure 7A). Dysregulated glycine, alanine, and glutamine metabolisms mirror observations in plasma from HUA-induced rats. Those rats exhibited upregulated glycine and alanine levels alongside downregulated glutamine levels [48]. In contrast, the abnormal levels of the remaining amino acids have not been reported previously. Cystathionine, homoserine, threonine, 2-oxo-butanoate, and 3-methyl-2-oxopentanoate are upstream metabolites for the synthesis of isoleucine; thus, the decreased levels of these metabolites might lead to reduced isoleucine levels, which is also found to be dysregulated in kidney diseases such as DKDs [49] and CKDs [50]. Homocysteine is a thiol-containing amino acid derived from methionine [51], and abnormal homocysteine levels are associated with CKDs [52] and DKDs [53] as well as cardiovascular events in individuals with CKDs [54]. The molecular mechanisms of homocysteine-induced renal damage include oxidative stress, endoplasmic reticulum stress, inflammation, and epigenomic regulation [51]. Homocysteine harbors a highly reactive thiol group and undergoes rapid auto-oxidation in the presence of oxygen and metal ions (e.g., iron, copper). This process generates potent reactive oxygen species, including superoxide anions, hydrogen peroxide, and hydroxyl radicals [51]. Additionally, homocysteine upregulates monocyte chemoattractant protein 1 mRNA and protein expression in kidney glomerular mesangial cells, and this effect is dependent on NF-κB activation [55]. These data suggest that homocysteine accelerates the progression of kidney disease by inducing inflammatory responses. We hypothesize that the increased homocysteine levels in Uox-KO mice are causally linked to HUA-induced renal inflammation. Histidine is an essential dietary amino acid in humans and has various biological functions, including proton buffering, metal ion chelation, scavenging of reactive oxygen and nitrogen species, and erythropoiesis [56]. The decreased histidine levels in Uox-KO mice are consistent with those in CKD patients. In CKD, a reduced histidine level is associated with inflammation, oxidative stress, and increased mortality [57]. L-histidine exhibits inherent antioxidant properties and scavenges free radicals and chelates divalent metal ions. This confers cytoprotective effects in CKD-related anemia [58]. Its antioxidant actions also inhibit the secretion of pro-inflammatory cytokines (e.g., interleukin (IL)-6, IL-8, IL-1β, tumor necrosis factor (TNF)-α) [58]. Leucine is a branched-chain amino acid that serves as an essential precursor for protein synthesis and energy production [59]; the decreased leucine levels in Uox-KO mice might indicate increased protein synthesis used to repair damaged membrane protein structures.

The TRP metabolism was also disrupted in Uox-KO mice. Acetoacetyl-CoA, L-kynurenate, 3-hydroxyanthranilate, serotonin, and 5-HIAA levels were significantly elevated, and, conversely, quinolinate, picolinate, TRP, and indole levels were markedly reduced (Figure 7A). This TRP metabolic dysregulation aligns with a previous untargeted analysis that reported increased L-kynurenine, xanthurenic acid, and 5-HIAA levels in Uox-KO mice [20]. Serotonin, also known as 5-hydroxytryptamine (5-HT), acts as a hormone, a neurotransmitter, and a mitogen and is ubiquitous across the animal kingdom [60]. Elevated serotonin levels were also observed in the plasma of patients with DKD [61]. Treatment with 5-HT receptor antagonists effectively prevents DKD [62,63]. Serotonin also regulates renal hemodynamics. For example, treating rat kidneys with a 5-HT2-receptor blocker increases blood flow and glomerular filtration rates [64]. We hypothesize that the increased serotonin levels in Uox-KO mice indicate dysregulated renal hemodynamics. 5-HIAA is a downstream metabolite of serotonin; therefore, the increased 5-HIAA levels in the kidneys might be caused by the increased serotonin levels in Uox-KO mice. TRP is an essential amino acid required for the synthesis of proteins and several important bioactive molecules. The decreased TRP levels in Uox-KO mice are consistent with studies related to AKI and CKD [65,66]. HUA might influence the TRP metabolism by inhibiting multidrug resistance protein 4 and the breast cancer resistance protein [67]. Therefore, we speculate that such a mechanism might exist, which could account for the disturbance in the Uox-KO mice’s TRP metabolism.

Glycerophospholipids are essential structural components of cell membranes, playing key roles in cell signaling, membrane anchoring, and substrate transport [68].

Dysregulated glycerophospholipid metabolism is linked to multiple diseases. These include coronary artery disease, asthma, and neurological disorders [69,70,71]. Abnormal glycerophospholipid metabolism has been reported in CKD patients and animal models [68,72]. It is also observed in obstructive uropathy [73] and IgAN [30]. For example, patients with CKDs display significantly elevated serum levels of total free fatty acids, glycerolipids, and glycerophospholipids. These lipids are positively associated with serum triglyceride levels and negatively associated with total cholesterol levels and the estimated glomerular filtration rate [68]. Similarly, plasma glycerophospholipid levels are dramatically increased in IgAN patients compared to healthy controls. Affected lipids include phosphatidylcholine, phosphatidylethanolamine, phosphatidylglycerol, phosphatidylinositol, and phosphatidylserine [30]. In this study, we report abnormal glycerophospholipid metabolism in Uox-KO mouse kidneys for the first time. The levels of glycerone-P, sn-glycerol-3P, sn-glycero-3-phosphochine, CDP-choline, phospho-choline, phosphatidyl-ethanolamine, CDP-ethanolamine, and phospho-ethanolamine were significantly elevated, and phosphatidylcholine and ethanol-amine levels were decreased (Figure 7B). This is consistent with findings in human HUA and HUA mouse models induced via potassium oxonate with or without UA [74]. Glycerophospholipid metabolism disorders might be closely linked with renal fibrosis processes in CKD and obstructive uropathy [73,75,76,77]. The aberrant expression of key metabolites in glycerophospholipid metabolism was detected in a rat renal fibrosis model established via a subcutaneous corticosterone injection. Notably, the levels of phosphatidylethanolamine and multiple phosphocholines were markedly reduced, indicating that these metabolites are potentially involved in the progression of renal fibrosis [75]. As renal interstitial fibrosis is a major phenotype of Uox-KO mice (Figure 1L), we speculate that the altered levels of these metabolites in the glycerophospholipid metabolism pathway in Uox-KO mice might be involved in renal interstitial fibrosis processes.

We simultaneously identified dysregulation in serotonergic synapse metabolism. cAMP, 5-HIAA, and serotonin levels were markedly elevated, and TRP, 8,9-EET, 11,12-EET, 14,15-EET, 5,6-DHET, 11,12-DHET, and 14,15-DHET levels were reduced (Figure 7C). 8,9-EET, 11,12-EET, 14,15-EET, 5,6-DHET, 11,12-DHET, and 14,15-DHET are metabolic products of arachidonic acid (AA). AA, also called eicosa-5,8,11,14-tetraenoic acid, is a ω-6 polyunsaturated fatty acid and a major component of cell membrane lipids [78,79]. AA can be metabolized by three distinct enzyme systems, namely cyclooxygenase, lipoxygenase, and cytochrome P450 enzymes. Abnormal AA metabolism (AAM) is closely related to several types of human diseases, including hypertension, obesity, diabetes, non-alcoholic fatty liver, asthma, cancer, and cardiovascular disease [79,80]. In this study, we detected AAM disturbances in the kidneys of Uox-KO mice, with dramatically decreased levels of 8,9-, 11,12-, and 14,15-EET and 5,6-, 11,12-, and 14,15-DHET (Figure 7C). The kidney has a high capacity to generate EETs (5,6-, 8,9-, 11,12-, and 14,15-EET), and these metabolites regulate epithelial cell transport and renal hemodynamics [81]. In addition, specific renal functions have been identified for each EET. 8,9-EET can dose-dependently prevent the focal and segmental glomerulosclerosis permeability factor-induced increase in glomerular albumin permeability in rats. Thus, 8,9-EET protects the glomerular filtration barrier. This barrier comprises the capillary endothelial cells, basement membrane, and podocyte slit membranes and selectively filters plasma components [82]. The decreased 8,9-EET levels in Uox-KO mice may indicate weakened glomerular barrier protection. This aligns with the abnormal glomerular phenotypes observed via histopathological analysis. 11,12-EET and 14,15-EET have been reported to be involved in hypertension, cardiovascular disease, and inflammatory processes in several types of renal diseases [78,83,84]. 11,12-EET is a major catalytic product of Cyp2c44 in mice. It regulates epithelial sodium excretion in the renal collecting duct. Accumulating evidence indicates that 11,12-EET may indirectly modulate renal inflammation via blood pressure regulation [85]. 14,15-EET ameliorates cyclosporine-induced proteinuria and renal dysfunction. This effect is linked to suppressed inflammatory cell infiltration in renal tissues and also involves mitigated renal fibrosis [86]. In a mouse model of ischemia/reperfusion -induced AKI, an exogenous 14,15-EET treatment attenuated renal tubular dilation and significantly decreased plasma levels of CRE, TNF-α, and IL-6 [87]. Moreover, 14,15-EET activates endothelial nitric oxide synthase and promotes NO release by inhibiting Ca^2+^-activated K^+^ channels, thereby inducing afferent arteriole dilation and ultimately alleviating renal inflammation [88]. In addition, the endogenous anti-fibrotic capacity of 14,15-EET has been recently verified in a unilateral ureteral obstruction mouse model. The supplemental administration of 14,15-EET markedly reduced the collagen-positive area, hydroxyproline content, and α-SMA-positive region in kidney tissues [89]. This protective mechanism is mediated by the inhibition of the renal epithelial–mesenchymal transition (EMT), as the expression of key EMT inducers (e.g., Snail1 and ZEB1) was notably downregulated after the 14,15-EET intervention [89]. We speculate that the decreased 11,12- and 14,15-EET levels in Uox-KO mice might be consistent with interstitial fibrosis and reflect the inflammatory status of the kidneys, as described previously [20,32].

Once formed, EETs are further hydrolyzed by soluble epoxide hydrolase (sEH). This reaction produces corresponding DHETs, which are considered inactivation products of EETs [78,83]. The obviously decreased DHET levels in Uox-KO mice have also been observed in patients with DKD [90,91]. For example, plasma concentrations of 14,15-DHET and 11,12-DHET were significantly lower in patients with DKD than in non-diabetic individuals [90]. This finding is consistent with the renal metabolic abnormalities observed in our Uox-KO mouse model. We speculate that the decreased DHET levels might be caused by reduced EET levels, as DHETs are downstream metabolites of EETs directly catalyzed by sEH.

To identify candidate metabolic biomarkers for CUN, we performed an AUC analysis of the differential metabolites in the kidneys. A total of 11 metabolites were identified via classical univariate ROC analyses with an AUC = 1, including pyroglutamic acid, fructose, riboflavin, dimethyl-L-arginine, glucaric acid, indoxyl sulfate, palmitoylethanolamide, trimethylamine N-oxide, 3-hydroxyanthranilic acid, spermidine, and hippuric acid. A correlation analysis revealed that all these metabolites were closely associated with renal function parameters. Collectively, these findings suggest that these metabolites might be potential predictors of CUN.

This study has several limitations that should be acknowledged. First, the sample size was relatively small, with only *n* = 6 mice per group for both Uox-KO and WT cohorts. This sample size is marginal for robust multivariate statistical analyses, including OPLS-DA modeling, VIP-driven metabolite selection, and correlation analyses between differential metabolites and renal functional parameters, which may reduce the statistical power of the observations. Second, no formal statistical power calculation was performed prior to the experiment to determine the optimal sample size for metabolomic profiling, which represents a methodological limitation. Third, despite strict QC procedures, limited analytical variation in QC samples, and rigorous data filtering (VIP > 1, *p* < 0.05), the small sample size still carries an inherent risk of false positive discoveries in the identification of differential metabolites and enriched pathways. Fourth, no independent external validation cohort was included in this study; thus, the use of the 11 candidate renal metabolic biomarkers for the prediction of CUN still requires further verification in larger independent animal cohorts. Finally, all experimental animals were age- and sex-matched but not strictly controlled as littermates, and potential variations from independent litters might introduce minor confounding effects on metabolic phenotypes. These limitations indicate that the present findings should be interpreted with appropriate caution, and further studies with larger sample sizes, power analysis, independent validation, and littermate-controlled designs are warranted to confirm the robustness of the metabolic signatures and biomarker candidates.

## 5. Conclusions

In this study, we employed MRM-based targeted metabolomics to characterize the renal metabolic landscape in a CUN mouse model, which was established via the knockout of the Uox gene. Our analyses revealed widespread perturbations in metabolite abundances within the kidneys of Uox-KO mice. Functional enrichment mapping demonstrated that these differential metabolites were significantly enriched in key metabolic and signaling pathways, including the biosynthesis of amino acids, ABC transporters, purine metabolism, aminoacyl-tRNA biosynthesis, protein digestion and absorption, glycerophospholipid metabolism, and serotonergic synapse. Furthermore, via a classical univariate ROC analysis, we identified 11 metabolite candidates with perfect discriminatory power (AUC = 1), suggesting their potential utility as non-invasive putative predictors for CUN. Correlation studies further substantiated the strong association between these metabolite panels and critical renal function parameters, reinforcing their clinical relevance. Collectively, this work validates targeted metabolomic analysis as a powerful and insightful strategy for unraveling the complex molecular mechanisms underlying CUN. The identification of key perturbed pathways and candidate biomarkers not only deepens our current understanding of the CUN pathogenesis but also offers novel translational perspectives for the development of targeted therapeutic interventions for this condition.

## Figures and Tables

**Table 1:** Potential metabolic biomarkers for chronic urate nephropathy in Uox-KO mice.

Metabolite Name	VIP	Fold-Change	*p*-Value	Compound Class	Label	Confidence Level
Pyroglutamic acid	1.6018337	0.28835	2.11 × 10^−6^	Amino acids, peptides, and analogs	down	Level 1
Fructose	1.5954118	2.6474	4.68 × 10^−7^	Sugar and derivatives	up	Level 1
Riboflavin	1.5712089	0.43611	1.05 × 10^−5^	Indoles and heterocyclic compounds	down	Level 1
Dimethyl-L-arginine	1.5542553	0.37179	3.44 × 10^−5^	Amino acids, peptides, and analogs	down	Level 2
Glucaric acid	1.4796976	5.8869	0.0001739	Organic acids and derivatives	up	Level 2
Indoxyl sulfate	1.4441036	5.7386	0.0001343	Indoles and heterocyclic compounds	up	Level 1
Palmitoylethanolamide	1.3687033	0.40007	0.0004409	Amines, choline, and organonitrogen compounds	down	Level 2
Trimethylamine N-oxide	1.3166437	9.0069	0.0012998	Amines, choline, and organonitrogen compounds	up	Level 2
3-Hydroxyanthranilic acid	1.2637099	4.8648	0.0024075	Organic acids and derivatives	up	Level 1
Spermidine	1.2554446	3.1751	0.0050288	Amines, choline, and organonitrogen compounds	up	Level 2
Hippuric acid	1.1131107	15.761	0.022992	Organic acids and derivatives	up	Level 1

Average values were calculated from six Uox-KO mice and six WT mice. Confidence level: Level 1: metabolites with perfect matches of retention time (RT), MS1, and MS2 spectra against the in-house library; Level 2: metabolites with perfect matches of MS1 and MS2 spectra against the in-house library.

**Table 2:** Correlations of the differential metabolites with renal function parameters.

Metabolites	UA	CRE	BUN	TIS
pyroglutamic acid	−0.713 *	−0.796 **	−0.741 **	−0.916 ***
fructose	0.797 **	0.663 *	0.797 **	0.756 **
riboflavin	−0.832 **	−0.818 **	−0.839 **	−0.869 **
dimethyl-L-arginine	−0.797 **	−0.782 **	−0.769 **	−0.869 **
glucaric acid	0.769 **	0.811 **	0.762 **	0.869 **
indoxyl sulfate	0.797 **	0.828 **	0.797 **	0.869 **
palmitoylethanolamide	−0.706 *	−0.737 **	−0.699 *	−0.869 **
trimethylamine N-oxide	0.734 **	0.726 *	0.748 **	0.869 **
3-hydroxyanthranilic acid	0.79 **	0.779 **	0.832 **	0.869 **
spermidine	0.692 *	0.74 **	0.699 *	0.869 **
hippuric acid	0.755 **	0.789 **	0.734 **	0.869 **

UA: uric acid; CRE: creatinine; BUN: urea nitrogen; TIS: tubular injury score. * *p* < 0.05; ** *p* < 0.01; *** *p* < 0.001.

## Data Availability

The original contributions presented in this study are included in the article/supplementary material. Further inquiries can be directed to the corresponding author.

## Supplementary Materials

The following supporting information can be downloaded at: https://www.mdpi.com/article/10.3390/metabo16060362/s1, Figure S1: The base peak chromatogram (BPC) of all the QC samples; Figure S2: Coefficient of variation (CV) distributions of compounds in each group of samples. Table S1: Differentially expressed kidney metabolites in Uox-Ko vs. WT mice in the ESI+ mode; Table S2: Differentially expressed kidney metabolites in Uox-Ko vs. WT mice in the ESI– mode.

## Abbreviations

The following abbreviations are used in this manuscript:

UA	uric acid
HUA	hyperuricemia
CKD	chronic kidney disease
DKD	diabetes kidney disease
AKI	acute kidney injury
MRM	multiple reaction monitoring
IgAN	immunoglobulin A nephropathy
UN	urate nephropathy
CUN	chronic urate nephropathy
KO	knockout
Uox	urate oxidase
WT	wild type
PCR	polymerase chain reaction
CRE	creatinine
BUN	urea nitrogen
GLU	glucose
TG	triglycerides
TC	total cholesterol
ALT	alanine aminotransferase
AST	aspertate aminotransferase
HDL	high-density lipoprotein
LDL	low-density lipoprotein
ALB	albumin
H & E	Hematoxylin-eosin
MT	Masson’s trichrome
TIS	tubular injury score
LC-MS	liquid chromatography-mass spectrometry
QC	quality control
UPLC	ultraperformance liquid chromatography
ESI	electrospray ionization
RT	retention time
PCA	principle component analysis
PLS-DA	partial least squares discriminant analysis
VIP	variable important for the projection
cAMP	3′, 5′-cyclic AMP
KEGG	Kyoto Encyclopedia of Genes and Genomes
ROC	receiver operating characteristic
AUC	area under the curve
CV	coefficient of variation
FA	fatty acids
LPE	lysophosphatidylethanolamines
TRP	Tryptophan
5-HIAA	5-OH-indoleacetate
EET	epoxyeicosatrienoic acids
DHET	dihydroxyeicosatrienoic acids
TCA	tricarboxylic acid
IL	interleukin
TNF	tumor necrosis factor
5-HT	5-hydroxytryptamine
AA	arachidonic acid
AAM	arachidonic acid metabolism
EMT	epithelial–mesenchymal transition
sEH	soluble epoxide hydrolase
