# Supplementary material for: Multiple Reaction Monitoring (MRM)-Based Targeted Kidney Metabolite Profiling of a Mouse Model of Hyperuricemia
Source: Metabolites. 2026 May 27;16(6):362. doi: 10.3390/metabo16060362 (PMC13304198; doi:10.3390/metabo16060362)
Supplement: Supplementary file 1 [file metabolites-16-00362-s001.zip › metabolites-4261528-supplementary.pdf]

Supplementary Information

Multiple Reaction Monitoring (MRM)-Based Targeted Kidney Metabolite Profiling of a Mouse Model of Hyperuricemia

Hailong Li *, Tingting Tang, Qingli Zhang, Tingting Song, Zichu Zhao, Lei Zhu, Qu Chen, Haili Zhang, Yan Zhang and Jingjing Kong *

Department of Medicine, Qingdao Binhai University, 425 West Jialing River Road, Qingdao 266555, China; tangtingting@qdbhu.edu.cn (T.T.); zhangqingli@qdbhu.edu.cn (Q.Z.); songtingting@qdbhu.edu.cn (T.S.); zhaozichu@qdbhu.edu.cn (Z.Z.); zhulei@qdbhu.edu.cn (L.Z.); chenqu@qdbhu.edu.cn (Q.C.); zhanghaili@qdbhu.edu.cn (H.Z.); zhangyan@qdbhu.edu.cn (Y.Z.)

* Correspondence: lihailong@qdbhu.edu.cn (H.L.); kongjingjing@qdbhu.edu.cn (J.K.); Tel.: +86-532-85130915 (H.L.); +86-532-84120596 (J.K.)

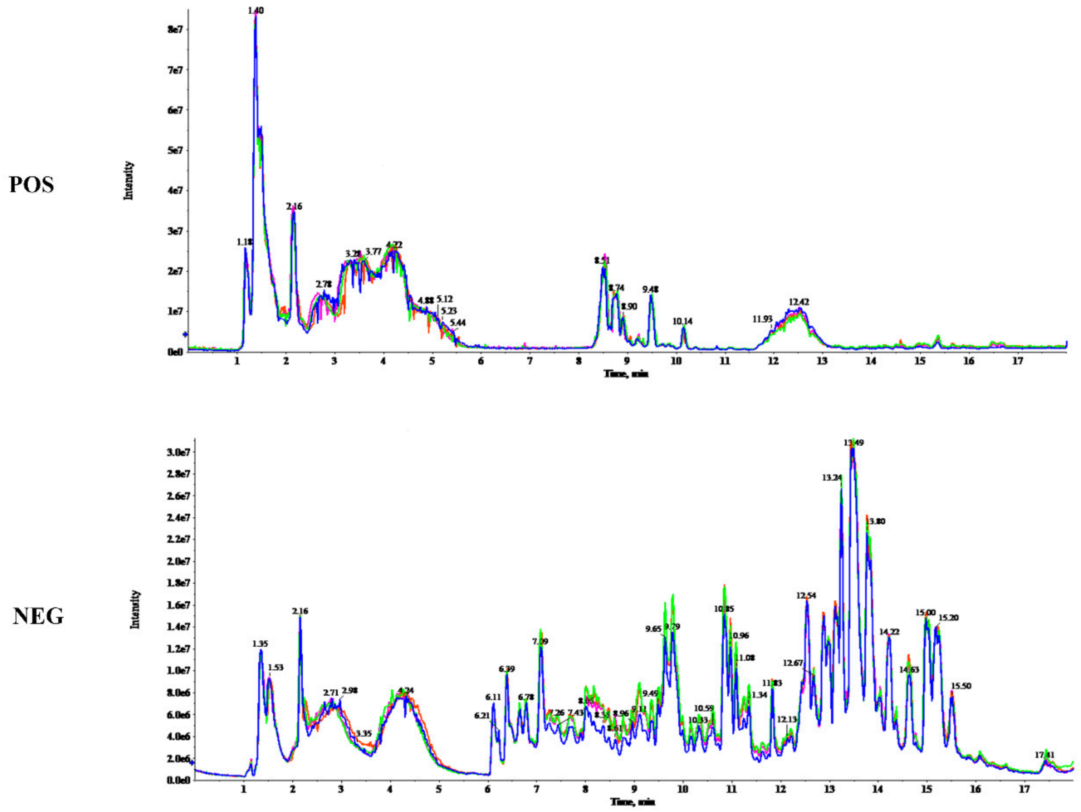

Figure S1. The base peak chromatogram (BPC) of all the QC samples. X-axis represents the retention time (min), Y-axis represents the intensity of MS (%). POS and NEG indicate the detections in the positive ion mode and negative ion mode, respectively.

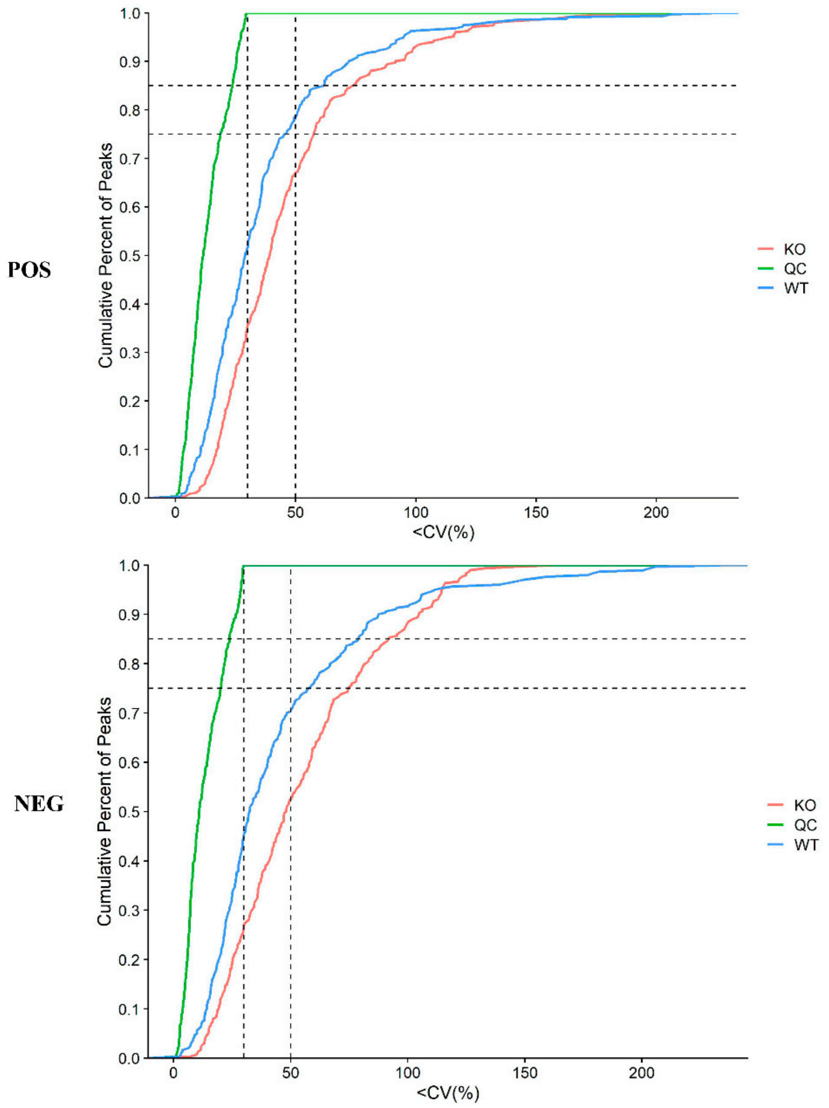

Figure S2. Coefficient of variation (CV) distributions of compounds in each group of samples. The two lines perpendicular to the X axis are the 30% and 50% CV reference lines, and the two lines parallel to the X axis are the reference lines accounting for 75% and 85% of peaks, respectively. POS and NEG indicate the detections in the positive ion mode and negative ion mode, respectively.

Table S1. Differentially-expressed kidney metabolites in Uox-KO vs. WT mice in the ESI+ mode.

Metabolite Name	VIP	Fold-Change	*p*-Value	Compound Class	Lable	Confidence Level
Riboflavin	1.5712089	0.43611	1.05E-05	Indoles and heterocyclic compounds	down	Level 1
Aspartate	1.5696115	0.56923	4.88E-06	Amino acids, peptides, and analogues	down	Level 1
L-Homoserine	1.5694051	0.54176	5.48E-06	Amino acids, peptides, and analogues	down	Level 1
Threonine	1.5655063	0.55567	6.58E-06	Amino acids, peptides, and analogues	down	Level 1
CMA	1.5635989	0.21822	8.64E-06	AGEs	down	Level 2
Dimethyl-L-arginine	1.5542553	0.37179	3.44E-05	Amino acids, peptides, and analogues	down	Level 2
N-alpha-Acetylarginine	1.54279	0.2145	3.99E-05	Amines,choline, and organonitrogen compounds	down	Level 2
Alanine	1.538902	0.71607	1.23E-05	Amino acids, peptides, and analogues	down	Level 1
Lactamide	1.5352438	0.63945	1.67E-05	Amines,choline, and organonitrogen compounds	down	Level 2
Saccharopine	1.5272604	0.57595	4.76E-06	Organic acids and derivatives	down	Level 2
cAMP	1.5248585	2.0414	7.68E-05	nucleosides,nucleotides, and analogues	up	Level 1
Serine	1.5225032	0.6224	8.05E-05	Amino acids, peptides, and analogues	down	Level 1
NG,NG-Dimethyl-L-arginine	1.5166712	0.38681	0.000128323	Amino acids, peptides, and analogues	down	Level 3
Sarcosine	1.5110561	0.68674	4.04E-05	Amino acids, peptides, and analogues	down	Level 2
3-Methyl-2-oxovaleric acid	1.4963402	0.52398	8.33E-05	Amino acids, peptides, and analogues	down	Level 3
Imidazoleacetic acid	1.4791892	2.0824	0.000193206	Organic acids and derivatives	up	Level 2
Glycerophosphocholine	1.4776185	4.6886	0.00011969	Acyl glycerol	up	Level 2
5-Aminopentanoic acid	1.475151	0.57798	0.000172355	Amino acids, peptides, and analogues	down	Level 2
Asparagine	1.4746991	0.68921	0.000348073	Amino acids, peptides, and analogues	down	Level 1
3-Aminoisobutyric acid	1.4659506	0.26479	0.000242629	nucleosides,nucleotides, and analogues	down	Level 2
L-Cystathionine	1.4635994	0.37844	0.000431465	Amino acids, peptides, and analogues	down	Level 2
N-Acetyl-L-leucine	1.4633619	0.43073	0.000174319	Amino acids, peptides, and analogues	down	Level 2
Deoxycarnitine	1.4566787	1.6399	0.000168317	Amines,choline, and organonitrogen compounds	up	Level 2

Metabolite Name	VIP	Fold-Change	p-Value	Compound Class	Lable	Confidence Level
Creatinine	1.456272	4.3225	0.000214065	Amino acids, peptides, and analogues	up	Level 1
Quinolin-2,8-diol	1.4558778	6.3596	0.000266916	Indoles and heterocyclic compounds	up	Level 3
5-Aminolevulinate	1.4551109	0.44114	0.000243241	Amino acids, peptides, and analogues	down	Level 1
Isopentenyladenosine	1.455102	0.35075	0.000198928	nucleosides,nucleotides, and analogues	down	Level 3
Oleoylethanolamide	1.4537255	0.22181	0.000127727	Amines,choline, and organonitrogen compounds	down	Level 2
O-Phosphoethanolamine	1.4453053	12.061	0.000372892	Amines,choline, and organonitrogen compounds	up	Level 2
SM 40:2;3	1.4432458	1.7735	0.000102772	Sphingolipids	up	Level 3
Arginine	1.4393539	0.6374	0.000300073	Amino acids, peptides, and analogues	down	Level 1
Dopamine	1.4383398	0.79427	0.000863515	Amines,choline, and organonitrogen compounds	down	Level 1
Purine	1.4381653	0.52293	0.000360008	nucleosides,nucleotides, and analogues	down	Level 2
3-Methyluridine	1.4366541	0.47502	0.000484693	nucleosides,nucleotides, and analogues	down	Level 2
2-Piperidone	1.435095	1.8251	0.000966149	Indoles and heterocyclic compounds	up	Level 2
Gamma-Aminobutyric acid	1.4348733	0.225	0.000412069	Amino acids, peptides, and analogues	down	Level 2
Kynurenic acid	1.4335175	7.2306	0.000293491	Amino acids, peptides, and analogues	up	Level 1
Hordenine	1.4297776	0.54031	0.000385384	Flavonoids, benzene and substituted derivatives	down	Level 1
1alpha,25-Dihydroxyvitamin D3	1.4270706	1.6344	0.000230911	Vitamins and derivatives	up	Level 3
Methionine	1.4260861	0.60458	0.000495523	Amino acids, peptides, and analogues	down	Level 1
Glycine	1.4194523	13.542	0.000208747	Amino acids, peptides, and analogues	up	Level 1
Aminohippuric acid	1.4186831	10.721	0.000429998	Flavonoids, benzene and substituted derivatives	up	Level 1
GABA	1.4177077	0.33774	0.000672343	Amino acids, peptides, and analogues	down	Level 2
Valine	1.4165155	0.65512	0.000479418	Amino acids, peptides, and analogues	down	Level 1
3-formylindole	1.415001	0.49816	0.00084821	Indoles and heterocyclic compounds	down	Level 2

Metabolite Name	VIP	Fold-Change	*p*-Value	Compound Class	Lable	Confidence Level
N6-methyladenosine	1.4125227	0.52683	0.000667876	nucleosides,nucleotides, and analogues	down	Level 2
1-Pyrroline-5-carboxylate	1.4098437	0.30699	0.001360725	Organic acids and derivatives	down	Level 3
Pantothenic acid	1.4071131	0.6005	0.000510635	Vitamins and derivatives	down	Level 2
N-Methylglutamic acid	1.396984	1.8997	0.000545382	Amino acids, peptides, and analogues	up	Level 3
N-Acetylornithine	1.3933714	0.50954	0.000661478	Amino acids, peptides, and analogues	down	Level 1
2'-O-methyladenosine	1.3914394	0.38171	0.000490884	nucleosides,nucleotides, and analogues	down	Level 2
N-Methyl-L-ornithine monohydrochloride	1.3879805	0.61163	0.001038612	Amino acids, peptides, and analogues	down	Level 3
Carnitine-C2	1.3872786	1.8437	0.000812158	Acyl carnitines	up	Level 2
Guanidinoacetic acid	1.3871025	0.74359	0.000864054	Amino acids, peptides, and analogues	down	Level 2
Xanthosine	1.3809943	0.61744	0.001227066	nucleosides,nucleotides, and analogues	down	Level 1
Indole	1.3801741	0.54387	0.001277279	Indoles and heterocyclic compounds	down	Level 1
Imidazole	1.3798858	1.9908	0.000827481	Indoles and heterocyclic compounds	up	Level 1
Anandamide	1.378487	0.3138	0.002138225	Amines,choline, and organonitrogen compounds	down	Level 1
Histidine	1.378482	0.62429	0.000735359	Amino acids, peptides, and analogues	down	Level 1
Ectoine	1.3762588	10.32	0.001621074	nucleosides,nucleotides, and analogues	up	Level 2
Palmitoylethanolamide	1.3687033	0.40007	0.000440888	Amines,choline, and organonitrogen compounds	down	Level 2
Proline	1.3631645	0.7456	0.00150738	Amino acids, peptides, and analogues	down	Level 2
7-methylguanine	1.3616648	0.65471	0.001461911	nucleosides,nucleotides, and analogues	down	Level 2
Tryptophan	1.3546772	0.76543	0.002604099	Amino acids, peptides, and analogues	down	Level 1
N-Acetyl-L-phenylalanine	1.349637	0.57932	0.001828649	Amino acids, peptides, and analogues	down	Level 3
SM 34:1;4	1.3327653	0.4325	0.004056326	Sphingolipids	down	Level 2
N-Acetyl-L-tyrosine	1.3258208	0.38826	0.003029719	Amino acids, peptides, and analogues	down	Level 2
Phosphorylcholine	1.3253607	10.435	0.002413396	Amines,choline, and organonitrogen compounds	up	Level 2

Metabolite Name	VIP	Fold-Change	*p*-Value	Compound Class	Lable	Confidence Level
CML	1.3223036	0.53476	0.003480421	AGEs	down	Level 2
gamma-Glutamyltyrosine	1.3172893	0.56946	0.001599324	Amino acids, peptides, and analogues	down	Level 2
Trimethylamine N-oxide	1.3166437	9.0069	0.001299834	Amines,choline, and organonitrogen compounds	up	Level 2
5-Hydroxyindoleacetate	1.3134229	11.965	0.003229343	Indoles and heterocyclic compounds	up	Level 1
N-Acetylhistamine	1.311112	2.4227	0.002861274	Amines,choline, and organonitrogen compounds	up	Level 2
Acetoacetyl-CoA	1.3056659	5.8344	0.002615697	acyl-CoAs	up	Level 2
Phenylacetylglycine	1.3009255	2.513	0.005266305	Amino acids, peptides, and analogues	up	Level 2
SM 38:0;2	1.2999527	0.53568	0.005231284	Sphingolipids	down	Level 2
Carnitine-C4	1.2971276	1.9586	0.004817428	Acyl carnitines	up	Level 2
Cytidine diphosphate choline	1.2960558	8.2089	0.003241595	nucleosides,nucleotides, and analogues	up	Level 2
Agmatine	1.2899962	1.5227	0.005322518	Amines,choline, and organonitrogen compounds	up	Level 1
D-Glucosamine	1.2861545	13.369	0.004559496	Sugar and derivatives	up	Level 2
3,4-dihydroxyphenylalanine	1.2816148	1.7953	0.009189565	Amines,choline, and organonitrogen compounds	up	Level 2
Cystine	1.2810113	3.2775	0.004163497	Amino acids, peptides, and analogues	up	Level 2
Isovalerylcarnitine	1.2791348	2.2599	0.003137528	Acyl carnitines	up	Level 2
Carnitine-C10:2	1.2758378	0.20443	0.007029679	Acyl carnitines	down	Level 2
Urocanic acid	1.2753667	4.4898	0.004044654	Indoles and heterocyclic compounds	up	Level 1
Lysine	1.2753502	0.73977	0.007362214	Amino acids, peptides, and analogues	down	Level 1
SM 44:1;3	1.2696246	1.7928	0.002712962	Sphingolipids	up	Level 2
Ethanolamine	1.2680121	0.58193	0.004791798	Amines,choline, and organonitrogen compounds	down	Level 2
SM 42:1;2	1.2662735	1.9307	0.003854777	Sphingolipids	up	Level 2
Carnitine-C10 OH	1.2658317	0.32372	0.007195069	Acyl carnitines	down	Level 2

Metabolite Name	VIP	Fold-Change	*p*-Value	Compound Class	Lable	Confidence Level
3-Hydroxyanthranilic acid	1.2637099	4.8648	0.002407464	Organic acids and derivatives	up	Level 1
2-Methylindole	1.2618822	0.55744	0.005797894	Indoles and heterocyclic compounds	down	Level 2
Glutamine	1.2597468	0.74126	0.00726875	Amino acids, peptides, and analogues	down	Level 1
1-Methyladenosine	1.2586675	0.58447	0.008509866	nucleosides,nucleotides, and analogues	down	Level 1
N1-Methyl-2-pyridone-5-carboxamide	1.2584336	4.4455	0.004766107	Indoles and heterocyclic compounds	up	Level 3
Biotin	1.2568735	0.36718	0.004043832	Vitamins and derivatives	down	Level 1
Spermidine	1.2554446	3.1751	0.005028815	Amines,choline, and organonitrogen compounds	up	Level 2
Carnitine-C5	1.2544156	2.2896	0.003791899	Acyl carnitines	up	Level 2
Nicotinamide	1.2491023	0.82527	0.00737686	Indoles and heterocyclic compounds	down	Level 2
SM 36:1;2	1.2478689	2.7995	0.007499002	Sphingolipids	up	Level 2
Quinic acid	1.2449079	4.6191	0.007423907	Organic acids and derivatives	up	Level 1
2-Hydroxyadenine	1.2412666	0.65255	0.013002949	nucleosides,nucleotides, and analogues	down	Level 2
Cortisol	1.2383632	0.3148	0.004863672	Steroids, hormones	down	Level 2
SM 40:3;3	1.2375804	1.8872	0.005948265	Sphingolipids	up	Level 3
5'-Deoxyadenosine	1.2321818	0.47985	0.010151343	nucleosides,nucleotides, and analogues	down	Level 2
Sepiapterin	1.2278339	0.41666	0.007762624	Indoles and heterocyclic compounds	down	Level 2
3-Methylcytidine	1.225778	1.6832	0.012088846	nucleosides,nucleotides, and analogues	up	Level 3
N6-glycerinyl	1.2242051	0.59863	0.007658152	AGEs	down	Level 3
L-Argininosuccinic acid	1.222112	0.53365	0.005517585	Amino acids, peptides, and analogues	down	Level 2
AMP	1.2164824	2.4422	0.006535531	nucleosides,nucleotides, and analogues	up	Level 2
Carnitine-C4DC	1.2057846	0.81253	0.019536602	Acyl carnitines	down	Level 3
CEA	1.2004572	0.59939	0.006900973	AGEs	down	Level 2
gamma-Glutamylleucine	1.1945877	0.51406	0.014539911	Amino acids, peptides, and analogues	down	Level 3
Leucine	1.1897693	0.8373	0.006116491	Amino acids, peptides, and analogues	down	Level 2
Pyrraline	1.1739356	16.834	0.015104437	AGEs	up	Level 2
Targinine	1.1732015	0.77165	0.011698589	Amino acids, peptides, and analogues	down	Level 2

Metabolite Name	VIP	Fold-Change	p-Value	Compound Class	Lable	Confidence Level
Ornithine	1.168745	0.53383	0.01479049	Amino acids, peptides, and analogues	down	Level 2
TG46:3-FA18:3	1.1676754	0.43768	0.010469213	Acyl glycerol	down	Level 3
7-methylguanosine	1.1601398	0.60141	0.013011108	nucleosides,nucleotides, and analogues	down	Level 3
Homoarginine	1.1600471	0.75332	0.012394404	Amino acids, peptides, and analogues	down	Level 2
L-Homocysteine	1.1598579	3.3395	0.014840663	Amino acids, peptides, and analogues	up	Level 2
Betaine	1.1563469	0.69409	0.011683888	Amino acids, peptides, and analogues	down	Level 2
gamma-L-Glutamyl-L-phenylalanine	1.1509859	0.52843	0.015235872	Amino acids, peptides, and analogues	down	Level 3
SM 42:2;3	1.1506197	1.7758	0.007443997	Sphingolipids	up	Level 2
Oxypurinol	1.1498423	0.6637	0.020167466	nucleosides,nucleotides, and analogues	down	Level 2
S-Carboxymethyl-L-cysteine	1.1493054	1.5344	0.015613807	Amino acids, peptides, and analogues	up	Level 3
Cer d18:1/20:1	1.1471063	0.55166	0.010284608	Sphingolipids	down	Level 2
4-Aminobenzoic acid	1.1426685	1.4686	0.01358345	Vitamins and derivatives	up	Level 1
Thiazolidine-4-carboxylic acid	1.1411204	3.0057	0.017466416	Amino acids, peptides, and analogues	up	Level 3
beta-D-Glucosamine	1.1393011	0.61092	0.021274336	Sugar and derivatives	down	Level 3
Pseudouridine	1.1332871	1.3327	0.021593448	nucleosides,nucleotides, and analogues	up	Level 2
Guanosine	1.1295297	0.74599	0.025053167	nucleosides,nucleotides, and analogues	down	Level 2
Nicotinic acid	1.1263247	0.82289	0.028114615	Indoles and heterocyclic compounds	down	Level 2
Picolinic acid	1.1259312	0.74936	0.02055564	Amino acids, peptides, and analogues	down	Level 2
N6-threonyl	1.1248123	2.3491	0.010074023	AGEs	up	Level 3
3-methoxytyramine	1.1158592	0.48764	0.017639448	Amines,choline, and organonitrogen compounds	down	Level 3
Urea	1.1130912	1.5719	0.013678246	Amino acids, peptides, and analogues	up	Level 2
TMP	1.1072867	12.696	0.019435286	nucleosides,nucleotides, and analogues	up	Level 2
Pyridine	1.1010774	0.75261	0.024543197	Indoles and heterocyclic compounds	down	Level 2
Beta-alanine	1.0992147	1.7731	0.024166593	Amino acids, peptides, and analogues	up	Level 2
L-O-Phosphoserine	1.0982788	1.4746	0.026292601	Amino acids, peptides, and analogues	up	Level 3
Carnitine-C16OH	1.0901961	1.8596	0.01503399	Acyl carnitines	up	Level 3

Metabolite Name	VIP	Fold-Change	*p*-Value	Compound Class	Lable	Confidence Level
Vitamin B1	1.0888688	0.72086	0.033818044	nucleosides,nucleotides, and analogues	down	Level 2
N-Fructosyl isoleucine	1.0745321	0.39968	0.032359373	Sugar and derivatives	down	Level 3
Epinephrine (E)	1.0723275	3.056	0.029160435	Amines,choline, and organonitrogen compounds	up	Level 2
Asp-Phe	1.066252	0.31648	0.01596571	Amino acids, peptides, and analogues	down	Level 2
Hydroxycholesterol species	1.0659745	0.33695	0.019638306	Steroids, hormones	down	Level 2
Serotonin	1.0585189	13.576	0.027780344	Indoles and heterocyclic compounds	up	Level 1
Hypotaurine	1.0581835	2.5227	0.02964649	Amino acids, peptides, and analogues	up	Level 1
5'-Methylthioadenosine	1.0447424	1.5079	0.019321279	nucleosides,nucleotides, and analogues	up	Level 3
4-Guanidinobutyric acid	1.0396428	1.2895	0.028847893	Organic acids and derivatives	up	Level 2
4-Methyl-2-oxovaleric acid	1.0382483	6.9668	0.032764761	Amino acids, peptides, and analogues	up	Level 3
2-Arachidonoylglycerol	1.0364929	0.42886	0.019960971	Acyl glycerol	down	Level 2
Hexanoylcarnitine	1.0215187	1.902	0.039006665	Acyl carnitines	up	Level 2
Deoxyinosine	1.014744	0.17392	0.018848532	nucleosides,nucleotides, and analogues	down	Level 2
3, 5-Tetradecadiencarnitine	1.0139536	0.64882	0.031316299	Acyl carnitines	down	Level 3
Cysteamine	1.0106941	2.501	0.025550737	Amino acids, peptides, and analogues	up	Level 2
Carnitine-C6	1.0080753	2.1058	0.039004729	Acyl carnitines	up	Level 2

Average values were calculated from six Uox-KO mice and six WT mice. Confidence level: Level 1: metabolites with perfect matches of retention time (RT), MS1, and MS2 spectra against the in-house library; Level 2: metabolites with perfect matches of MS1 and MS2 spectra against the in-house library; Level 3: metabolites with perfect matches of RT and MS1 against the in-house library.

Table S2. Differentially-expressed kidney metabolites in Uox-KO vs. WT mice in the ESI− mode.

Metabolite Name	VIP	Fold-Change	*p*-Value	Compound Class	Lable	Confidence Level
Pyrophosphate	1.609701	0.45202	1.59E-06	Organic acids and derivatives	down	Level 2
Gamma-glutamyl-Cysteine	1.603744	0.19567	8.19E-07	Amino acids, peptides, and analogues	down	Level 2
Pyroglutamic acid	1.601834	0.28835	2.11E-06	Amino acids, peptides, and analogues	down	Level 1
Fructose	1.595412	2.6474	4.68E-07	Sugar and derivatives	up	Level 1
Indole-3-carboxylic acid	1.588896	1.9036	6.77E-06	Organic acids and derivatives	up	Level 2
Adenylsuccinic acid	1.547185	0.26095	3.86E-06	nucleosides,nucleotides, and analogues	down	Level 2
Sedoheptulose monophosphate	1.543562	3.1566	1.36E-05	Sugar and derivatives	up	Level 2
L-Lactate	1.541059	1.9823	1.64E-05	Organic acids and derivatives	up	Level 2
FA(20:2)	1.541037	0.48531	1.96E-05	Fatty acids	down	Level 3
Uracil	1.530576	0.50561	8.26E-05	nucleosides,nucleotides, and analogues	down	Level 1
D-Glucose 6-phosphate	1.52068	4.1857	0.00014933	Sugar and derivatives	up	Level 2
FA(22:6)	1.519502	0.59332	4.30E-05	Fatty acids	down	Level 2
Carbamoyl phosphate	1.515958	19.183	7.21E-05	nucleosides,nucleotides, and analogues	up	Level 2
LPE(18:1)	1.509519	0.54276	5.57E-06	Phosphatidylacids	down	Level 2
LPE(22:6)	1.509164	0.52478	0.00013616	Phosphatidylacids	down	Level 2
Dihydroxyacetone phosphate	1.503413	3.917	6.47E-05	Organic acids and derivatives	up	Level 3
Adipic acid	1.502327	0.1986	6.04E-05	Organic acids and derivatives	down	Level 1
Indole-3-carboxaldehyde	1.501243	0.22478	3.93E-05	Indoles and heterocyclic compounds	down	Level 1
LPE(20:4)	1.498303	0.74481	0.00015828	Phosphatidylacids	down	Level 2
Acetoacetic acid	1.491844	0.21453	4.41E-05	Organic acids and derivatives	down	Level 1
Glucaric acid	1.479698	5.8869	0.00017388	Organic acids and derivatives	up	Level 2
Hydroxyisocaproic acid	1.475486	3.2124	0.00019474	Fatty acids	up	Level 2
FA(20:4)	1.460434	0.71905	0.00021629	Fatty acids	down	Level 2
2-Deoxyinosine triphosphate	1.45391	3.3496	0.00024251	nucleosides,nucleotides, and analogues	up	Level 2
N-Formylmethionine	1.453148	2.6727	0.00022231	Amino acids, peptides, and analogues	up	Level 2
Citrate	1.45051	2.5084	0.00016173	Organic acids and derivatives	up	Level 1

Metabolite Name	VIP	Fold-Change	*p*-Value	Compound Class	Lable	Confidence Level
Gamma-glutamyl-Alanine	1.45046	0.4767	0.00044938	Amino acids, peptides, and analogues	down	Level 1
4-Hydroxyquinoline	1.447632	0.2204	0.00018015	Indoles and heterocyclic compounds	down	Level 2
Glyceric acid	1.444437	0.30469	0.00035341	Sugar and derivatives	down	Level 1
Indoxyl sulfate	1.444104	5.7386	0.0001343	Indoles and heterocyclic compounds	up	Level 1
Glyceraldehyde 3-phosphate	1.438872	2.9436	0.00039442	Organic acids and derivatives	up	Level 2
Phosphoenolpyruvate	1.436056	10.097	0.00044665	Organic acids and derivatives	up	Level 2
Dehydroascorbic acid	1.431512	2.6736	0.00019231	Vitamins and derivatives	up	Level 2
L-Threonine	1.431276	0.43174	0.00035379	Amino acids, peptides, and analogues	down	Level 2
Threonate	1.429715	1.7632	0.00030165	Sugar and derivatives	up	Level 2
Dioxolithocholic acid	1.419121	0.60155	0.00061343	Bile acids	down	Level 1
FA(16:0)	1.414804	0.7691	0.00039358	Fatty acids	down	Level 2
FA(22:2)	1.413829	0.37162	0.00046485	Fatty acids	down	Level 2
Cholesterol sulfate	1.412498	0.29084	0.00045797	Steroids, hormones	down	Level 2
Heptadecanoic acid	1.409541	0.33377	0.0002551	Fatty acids	down	Level 1
Monoisobutyl phthalate	1.409052	2.1634	0.00078233	Flavonoids, benzene and substituted derivatives	up	Level 3
23-Nordeoxycholic acid	1.395322	0.19919	0.0005517	Bile acids	down	Level 3
Cinnamic acid	1.39056	1.8551	0.00086307	Flavonoids, benzene and substituted derivatives	up	Level 2
Acamprosate	1.387842	0.349	0.00070801	Organic acids and derivatives	down	Level 2
FA(12:0)	1.378962	0.32234	0.00049866	Fatty acids	down	Level 2
LPE(20:2)	1.370215	0.23582	0.00066584	Phosphatidylacids	down	Level 2
Homogentisic acid	1.368029	3.8315	0.00107127	Amino acids, peptides, and analogues	up	Level 2
FA(22:0)	1.367579	0.52848	0.00136278	Fatty acids	down	Level 2
Deoxyadenosine triphosphate	1.365368	6.3037	0.00084309	nucleosides,nucleotides, and analogues	up	Level 3
5-hydroxyeicosatatraeniocacid	1.365266	0.26391	0.00147721	Arachidonic acid metabolites	down	Level 2
8,9-Epoxyeicosatrienoic acid	1.362364	0.34618	0.00171561	Arachidonic acid metabolites	down	Level 2
Dodecylbenzenesulfonic acid	1.351494	0.50947	0.00118656	Flavonoids, benzene and substituted derivatives	down	Level 2
(9R)-9-Hydroxyeicosatetraenoic Acid	1.349674	0.33077	0.00193832	Arachidonic acid metabolites	down	Level 3

Metabolite Name	VIP	Fold-Change	*p*-Value	Compound Class	Lable	Confidence Level
FA(20:0)	1.346414	0.55526	0.00158114	Fatty acids	down	Level 2
Enterolactone	1.338971	2.1348	0.0019805	Steroids, hormones	up	Level 3
N-Acetylaspartylglutamate	1.337665	2.5752	0.00272504	Amino acids, peptides, and analogues	up	Level 2
2-Ketobutyric acid	1.335908	0.23428	0.00188988	Organic acids and derivatives	down	Level 2
2-Pyrocatechuic acid	1.330335	3.8819	0.0029125	Organic acids and derivatives	up	Level 2
Xanthine	1.327705	0.54276	0.00255112	nucleosides,nucleotides, and analogues	down	Level 1
Uric acid	1.325094	23.169	0.00123651	nucleosides,nucleotides, and analogues	up	Level 1
FA(16:2)	1.308969	0.62672	0.0032022	Fatty acids	down	Level 2
LPE(20:3)	1.305592	0.34632	0.00399054	Phosphatidylacids	down	Level 2
Octadecanedioic acid	1.29952	0.45952	0.00277014	Fatty acids	down	Level 1
Abietic acid	1.294293	0.47666	0.00477574	Organic acids and derivatives	down	Level 1
LPE(22:5)	1.292319	0.35126	0.00455772	Phosphatidylacids	down	Level 2
N-Acetyl-D-glucosamine 1-phosphate	1.292287	2.6258	0.00572265	Sugar and derivatives	up	Level 3
Pyridoxal 5-phosphate	1.290585	0.4643	0.00530183	Vitamins and derivatives	down	Level 1
2-Hydroxybenzoic acid	1.288212	2.0045	0.00539626	Flavonoids, benzene and substituted derivatives	up	Level 1
D-Erythrose 4-phosphate	1.285439	3.8822	0.00407856	Organic acids and derivatives	up	Level 2
D-Glucosamine 6-phosphate	1.281781	0.66044	0.00353585	Sugar and derivatives	down	Level 3
FA(20:3)	1.267582	0.69075	0.00753604	Fatty acids	down	Level 2
Guanosine 5'-diphospho-beta-L-fucose	1.266922	17.134	0.00590842	Sugar and derivatives	up	Level 3
LPE(22:4)	1.261017	0.44902	0.00449246	Phosphatidylacids	down	Level 2
Cytidine diphosphate ethanolamine	1.260262	6.868	0.00683681	nucleosides,nucleotides, and analogues	up	Level 2
8(R)-hydroxy- eicosatetraenoic acid	1.257182	0.38989	0.00682339	Arachidonic acid metabolites	down	Level 3
12 Dehydrocholic Acid sulfate	1.255308	0.48111	0.00372964	Bile acids	down	Level 2
3-phosphoglycerate	1.254999	10.501	0.00635798	Organic acids and derivatives	up	Level 1
Thiamin pyrophosphate	1.248296	1.9977	0.00609521	Vitamins and derivatives	up	Level 2
Eicosapentaenoic acid	1.227073	0.52167	0.00892334	Fatty acids	down	Level 1
14,15-Epoxy-5,8,11-eicosatrienoic acid	1.22354	0.40142	0.00796177	Arachidonic acid metabolites	down	Level 3

Metabolite Name	VIP	Fold-Change	*p*-Value	Compound Class	Lable	Confidence Level
Oxaloacetic acid	1.218622	0.64059	0.00438849	Organic acids and derivatives	down	Level 1
2,3-Dihydroxybenzoic acid	1.217405	6.1188	0.01254891	Flavonoids, benzene and substituted derivatives	up	Level 2
FA(18:1)	1.213095	0.75671	0.00631229	Fatty acids	down	Level 2
n-Capric acid	1.207386	0.47028	0.00346367	Fatty acids	down	Level 2
D-glucosamine 1-phosphate	1.204701	0.6395	0.00564885	Sugar and derivatives	down	Level 2
PG(16:0/18:1)	1.203021	0.68103	0.00832118	Phosphatidylacids	down	Level 2
Quinolinic Acid	1.186948	0.45163	0.00944102	Organic acids and derivatives	down	Level 1
FA(18:2)	1.186576	0.79662	0.00640472	Fatty acids	down	Level 2
FA(24:3)	1.182856	0.28348	0.00562345	Fatty acids	down	Level 2
beta-Hydroxymyristic acid	1.181461	0.54986	0.01165644	Fatty acids	down	Level 2
13,14-Dihydro-15-keto PGF2a	1.181358	0.16752	0.0073707	Arachidonic acid metabolites	down	Level 3
Phenylacetylglutamine	1.178651	0.72355	0.01076941	Amino acids, peptides, and analogues	down	Level 2
Orotidine monophosphate	1.178308	0.5506	0.0156198	nucleosides,nucleotides, and analogues	down	Level 2
15-Hydroxyeicosatetraenoic acid	1.176478	0.40298	0.01086092	Arachidonic acid metabolites	down	Level 2
LPE(18:2)	1.17423	0.5462	0.01070742	Phosphatidylacids	down	Level 2
Lipoic acid	1.17142	4.8891	0.01327429	Organic acids and derivatives	up	Level 1
LPE(18:3)	1.16448	0.52905	0.01612139	Phosphatidylacids	down	Level 2
PI(18:0/18:0)	1.162611	0.48898	0.01303435	Phosphatidylacids	down	Level 2
5,6-DHET	1.158379	0.30209	0.01149609	Arachidonic acid metabolites	down	Level 2
UDP-glucose	1.153753	21.929	0.01434708	nucleosides,nucleotides, and analogues	up	Level 1
UDP-glucuronate	1.151497	15.712	0.01482093	nucleosides,nucleotides, and analogues	up	Level 2
D-Glucuronic acid	1.143723	0.76479	0.02451044	Sugar and derivatives	down	Level 3
11-Hydroxyeicosatetraenoic acid	1.138604	0.49296	0.01799361	Arachidonic acid metabolites	down	Level 2
11,12-Epoxyeicosatrienoic acid	1.133255	0.47999	0.01755744	Arachidonic acid metabolites	down	Level 2
FA(18:3)	1.129818	0.67976	0.01693117	Fatty acids	down	Level 2
FA(18:0)	1.128061	0.56256	0.01381272	Fatty acids	down	Level 2
LPE(16:1)	1.126439	0.5484	0.01356066	Phosphatidylacids	down	Level 2

Metabolite Name	VIP	Fold-Change	*p*-Value	Compound Class	Lable	Confidence Level
Pentadecanoic acid	1.123058	0.55292	0.01078499	Fatty acids	down	Level 1
L-Dihydroorotic acid	1.119584	0.56536	0.02006113	Amino acids, peptides, and analogues	down	Level 1
Hippuric acid	1.113111	15.761	0.02299195	Organic acids and derivatives	up	Level 1
5-Hydroxy-6,8,11,14,17-eicosapentaenoic acid	1.112765	0.31689	0.01972489	Fatty acids	down	Level 3
4-Pyridoxic acid	1.109637	0.20722	0.01143186	Vitamins and derivatives	down	Level 2
Allantoin	1.107762	0.49342	0.02760829	nucleosides,nucleotides, and analogues	down	Level 1
Glycerol 3-phosphate	1.104439	3.1859	0.02541662	Acyl glycerol	up	Level 2
PG(18:0/18:1)	1.104372	0.22362	0.01230666	Phosphatidylacids	down	Level 2
FA(22:1)	1.099806	0.44863	0.01772607	Fatty acids	down	Level 2
11,12-DiHETrE	1.092424	0.30868	0.01774154	Arachidonic acid metabolites	down	Level 3
2'-Deoxyuridine 5'-triphosphate	1.08605	0.44667	0.0281587	nucleosides,nucleotides, and analogues	down	Level 3
PC(18:1/18:3)	1.073624	0.41122	0.03198575	Phosphatidylacids	down	Level 2
3-Indolepropionic acid	1.06573	0.6115	0.03498006	Indoles and heterocyclic compounds	down	Level 2
14,15-DHET	1.046495	0.34456	0.02688962	Arachidonic acid metabolites	down	Level 1
2-Hydroxy-3-methylbutyric acid	1.046344	2.4704	0.03518163	Organic acids and derivatives	up	Level 3
Cinnamoylglycine	1.043741	5.5488	0.03029752	Amino acids, peptides, and analogues	up	Level 2
Methylsuccinic Acid	1.043506	0.70826	0.0190264	Organic acids and derivatives	down	Level 2
3-Hydroxybenzaldehyde	1.041739	0.3885	0.02003704	Flavonoids, benzene and substituted derivatives	down	Level 2
FA(24:1)	1.032796	0.49612	0.02528003	Fatty acids	down	Level 2
PC(16:0/20:4)	1.025007	0.64383	0.04344311	Phosphatidylacids	down	Level 2
Pyruvate	1.024593	1.8172	0.01558328	Organic acids and derivatives	up	Level 1
Guanosine diphosphate	1.024563	1.942	0.0373387	nucleosides,nucleotides, and analogues	up	Level 1
PE(18:0/22:6)	1.021533	12.164	0.03390968	Phosphatidylacids	up	Level 2
Docosatetraenoic acid	1.01516	0.71764	0.03792383	Fatty acids	down	Level 1
Androsterone sulfate	1.009761	0.4688	0.04199804	Steroids, hormones	down	Level 1
FA(22:3)	1.007895	0.62211	0.03848623	Fatty acids	down	Level 2

Average values were calculated from six Uox-KO mice and six WT mice. Confidence level: Level 1: metabolites with perfect matches of retention time (RT), MS1, and MS2 spectra against the in-house library; Level 2: metabolites with perfect matches of MS1 and MS2 spectra against the in-house library; Level 3: metabolites with perfect matches of RT and MS1 against the in-house library.
